# ATAC-Seq identifies regions of open chromatin in the bronchial lymph nodes of dairy calves experimentally challenged with bovine respiratory syncytial virus

**DOI:** 10.1186/s12864-020-07268-5

**Published:** 2021-01-06

**Authors:** Dayle Johnston, JaeWoo Kim, Jeremy F. Taylor, Bernadette Earley, Matthew S. McCabe, Ken Lemon, Catherine Duffy, Michael McMenamy, S. Louise Cosby, Sinéad M. Waters

**Affiliations:** 1 Animal and Bioscience Research Department, Animal & Grassland Research and Innovation Centre, Teagasc, Grange, Co. Meath, Ireland; 2 Division of Animal Sciences, University of Missouri, Columbia, MO USA; 3 Veterinary Sciences Division, Agri-Food and Biosciences Institute, Stormont, Belfast, Northern Ireland

**Keywords:** ATAC-Seq, BRSV, Bovine respiratory disease, Dairy calves, Open chromatin, Gene regulation

## Abstract

**Background:**

Bovine Respiratory Syncytial Virus (BRSV) is a cause of Bovine Respiratory Disease (BRD). DNA-based biomarkers contributing to BRD resistance are potentially present in non-protein-coding regulatory regions of the genome, which can be determined using ATAC-Seq. The objectives of this study were to: (i) identify regions of open chromatin in DNA extracted from bronchial lymph nodes (BLN) of healthy dairy calves experimentally challenged with BRSV and compare them with those from non-challenged healthy control calves, (ii) elucidate the chromatin regions that were differentially or uniquely open in the BRSV challenged relative to control calves, and (iii) compare the genes found in regions proximal to the differentially open regions to the genes previously found to be differentially expressed in the BLN in response to BRSV and to previously identified BRD susceptibility loci. This was achieved by challenging clinically healthy Holstein-Friesian calves (mean age 143 ± 14 days) with either BRSV inoculum (*n* = 12) or with sterile phosphate buffered saline (PBS) (*n* = 6) and preparing and sequencing ATAC-Seq libraries from fresh BLN tissues.

**Results:**

Using Diffbind, 9,144 and 5,096 differentially accessible regions (*P* < 0.05, FDR < 0.05) were identified between BRSV challenged and control calves employing DeSeq2 and EdgeR, respectively. Additionally, 8,791 chromatin regions were found to be uniquely open in BRSV challenged calves. Seventy-six and 150 of the genes that were previously found to be differentially expressed using RNA-Seq, were located within 2 kb downstream of the differentially accessible regions, and of the regions uniquely open in BRSV challenged calves, respectively. Pathway analyses within ClusterProfiler indicated that these genes were involved in immune responses to infection and participated in the Th1 and Th2 pathways, pathogen recognition and the anti-viral response. There were 237 differentially accessible regions positioned within 40 previously identified BRD susceptibility loci.

**Conclusions:**

The identified open chromatin regions are likely to be involved in the regulatory response of gene transcription induced by infection with BRSV. Consequently, they may contain variants which impact resistance to BRD that could be used in breeding programmes to select healthier, more robust cattle.

**Supplementary Information:**

The online version contains supplementary material available at 10.1186/s12864-020-07268-5.

## Background

Rates of dairy calf mortality remain high globally, ranging from 5 to 11% [1]. In Ireland, the mortality rate for dairy calves between 0 and 6 months of age is 5.4% [2], while the pre-weaning dairy calf mortality rate in the US is 7.8% [3]. Bovine respiratory disease (BRD) accounts for the largest proportion of dairy calf mortality between 2 and 6 months of age [4]. The global prevalence of BRD in dairy calves varies greatly between studies, and ranges from 3.5 to 40% [5–10].

BRD is a disease of the upper and lower respiratory tract which results in the formation of syncytial cells in the bronchiolar epithelium and lung parenchyma, and clinical signs which include an elevated rectal temperature, increased respiratory rate, nasal and ocular discharges, cough, dyspnea, decreased appetite and depressive-like behaviour [11, 12]. Viral pathogens are generally responsible for the initiation of BRD and secondary bacterial pathogens, many of which are normally commensal in the nasopharyngeal region of the upper respiratory tract, often proliferate and exacerbate the disease [13–15].

Bovine respiratory syncytial virus (BRSV), an enveloped, negative-stranded RNA virus, is one of the primary infectious agents responsible for the onset of BRD [16, 17]. Despite BRD being a moderately heritable [18–20] multifactorial disease influenced by genetic predisposing factors, environmental conditions and husbandry management practices [10, 21], the available literature on the host genetic response to viral infections, including BRSV, is limited. An understanding of the identity of the variation within the bovine genome which confers variation in resistance to BRD is needed to incorporate genetic variants into breeding programmes designed to breed robust animals with increased resistance to BRD infection. In a previous study, we identified differentially expressed genes [22] and miRNAs (unpublished observations) in the bronchial lymph nodes (BLN) (the site of antigen presentation and activation of immune effector cells), of Holstein-Friesian calves experimentally challenged with BRSV. Additionally, the transcriptional response to infection with several pathogens involved in the bovine respiratory disease complex (BRDC) in BLN [23], lung and multiple lymphoid tissues [24] has previously been described in US Angus x Hereford crossbred beef steers. However, there is a lack of knowledge regarding the non-protein-coding regions of the genome which are involved in the regulation of the transcriptional response to BRD. Quantitative trait loci (QTL) and single nucleotide polymorphisms (SNPs) associated with BRD susceptibility [18, 20, 25–27] have been identified in dairy and beef cattle. Among these QTL, the genetic variants which are located in the regulatory regions that are actively involved in the host response to BRD, are most likely to be predictive of genetic merit for BRD resistance within and across cattle breeds. These active regulatory regions of the genome can be identified since the surrounding chromatin should be open and accessible by regulatory elements such as transcription factors.

Assay for Transposase-Accessible Chromatin using sequencing (ATAC-Seq) is a novel technique [28] used for the identification of regions of open chromatin (ROCs). Chromatin is open when it is in an uncondensed state (euchromatin) and is accessible to gene transcriptional machinery and DNA binding regulatory elements. When it is condensed and tightly wrapped around histone proteins (heterochromatin), it is in an inactive and transcriptionally inaccessible state [29]. While we have previously identified key genes that are expressed during BRSV infection [22], there is a lack of information on the specific regions of the genome that regulate the response to BRSV infection. The identification of the regions of chromatin that are open in respiratory tissues during BRSV infection will indicate the genomic regions that are transcriptionally active during infection. These regions may harbour DNA variants that affect the transcriptional immune response to BRSV and may allow the inference of genotypes with superior resistance to BRD. The objectives of the study were to: (i) identify regions of open chromatin in the BLN of dairy calves experimentally challenged with BRSV and also in control calves, (ii) elucidate the chromatin regions which were differentially or uniquely open in the BRSV challenged relative to the control calves, and (iii) compare the differentially open regions with the locations of genes previously found to be differentially expressed in the BLN in response to BRSV and with the locations of previously identified BRD susceptibility loci [18, 20, 25–27].

## Results

### Read quality, alignment and peak calling

ATAC-Seq libraries (*n* = 18) were prepared from fresh BLN tissue from BRSV challenged (*n* = 12) and control (*n* = 6) calves and sequenced on an Illumina NextSeq 500. An average (± SD) of 46,099,035 (± 8,156,367) (2 × 75 bp) paired-end reads (i.e., 23,049,517 sequenced fragments) were generated for each sample (Additional file 1). Approximately 96% of the reads were aligned to the UMD3.1 bovine reference genome assembly. Five percent of the reads mapped to the mitochondrial genome and 14% of the reads had a MAPQ score < 10. There were, on average, 4% of sequences that were duplicated among the non-mitochondrial sequences with a MAPQ score > 10. The average non-redundant fraction was 82%. However, two samples (calf numbers 4 and 5 from the control group) had considerably lower non-redundant fractions relative to the other samples, resulting in a higher percentage of samples with a MAPQ score < 10 (Additional file 1). This indicates that these samples contained a large number of reads which could be aligned to multiple places in the reference genome with equal stringency. An average of 33,140,167 (± 64,571) reads were used for peak calling in MACS2 following the removal of duplicate reads by MACS2 (Additional file 1).

There were more regions of open chromatin detected in the BLN of the BRSV challenged calves (39,105 ± 1479) than the control calves (29,094 ± 2422) (student’s T-test; *P* = 0.0019) (Additional file 2). The Bedtools Jaccard score was used to measure of the similarity of ROCs between two samples based on the ratio of the number of base pairs present in the intersection to the number present in the unique union of ROCs predicted for each sample. The mean Jaccard score (± SEM) for samples from control calves and BRSV challenged calves was 0.46 (± 0.025) and 0.59 (± 0.004), respectively (Additional file 2). Samples 4 and 5 from the control calves had lower Jaccard scores than the rest of the samples. Following removal of the Jaccard scores for these calves, the mean Jaccard score for the control calves increased to 0.54 (± 0.019).

### Diffbind analysis

The consensus peakset generated by Diffbind contained 57,504 ROCs, defined by overlapping ATAC-Seq reads across all samples. Fifty percent (28,635) of the ROCs were within 2 kb upstream of protein-coding (non-mitochondrial or Y chromosome) genes (Additional file 3). Ninety-three percent (26,518) of the ROCs within 2 kb upstream of a gene were closest to a gene expressed in the BLN (Additional file 3). Of the protein-coding genes expressed in the BLN [22], 82% (11, 047) had a ROC either within the gene or within 2 kb upstream of the gene. Twenty-two percent (1450) of the protein coding genes not expressed in the BLN had a ROC either within the gene or within 2 kb upstream of the gene. Forty-seven percent (27,061) of the ROCs were located within protein-coding genes (Additional file 3). Ninety-three percent (25,192) of ROCs located within protein-coding genes were closest to a gene expressed in the BLN (Additional file 3). Of the protein-coding genes expressed in the BLN [22], 80% (10,734) had a ROC within the gene body.

**Fig. 1:**
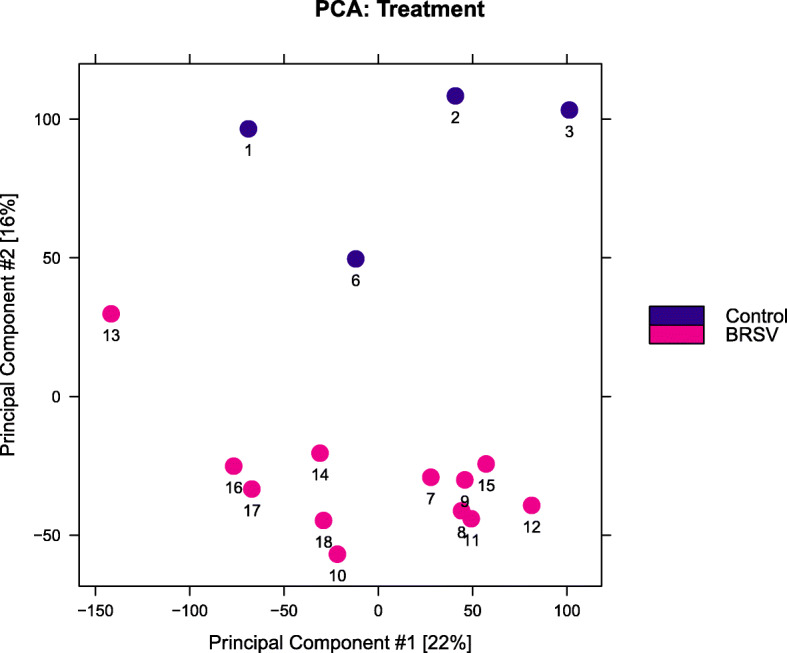
Principal component plot of bronchial lymph node ATAC-Seq regions of accessible chromatin (ROC) data. This plot was generated in Diffbind and illustrates the similarity of the BRSV challenged (*n* = 12) and control (*n* = 4) calves’ bronchial lymph node samples based on regions of accessible chromatin (ATAC-Seq ROCs). Bronchial lymph node tissue samples from BRSV challenged calves (Calf IDs 7 to 18) are coloured in pink and from control calves (Calf IDs 1, 2, 3 and 6) are coloured in purple

A principal component analysis (PCA) plot produced in Diffbind showed that calf ID samples 4 and 5 differed from all other samples (Additional file 4 (a)). These were the samples with lower library complexities indicated by their low non-redundant fractions. These samples were removed from all subsequent analyses and the new PCA plot produced revealed a separation between BRSV challenged and control calves on principal component (PC) 2 (Fig. 1, Additional file 4). The separation between samples on PC1 appeared to be caused by a combination of metrics determining library quality, including the percentage of reads which were properly paired and uniquely aligned, the percentage of reads with a MAPQ score less than 10, the percentage of mitochondrial reads and the quantity of library produced (Additional file 4).

**Fig. 2:**
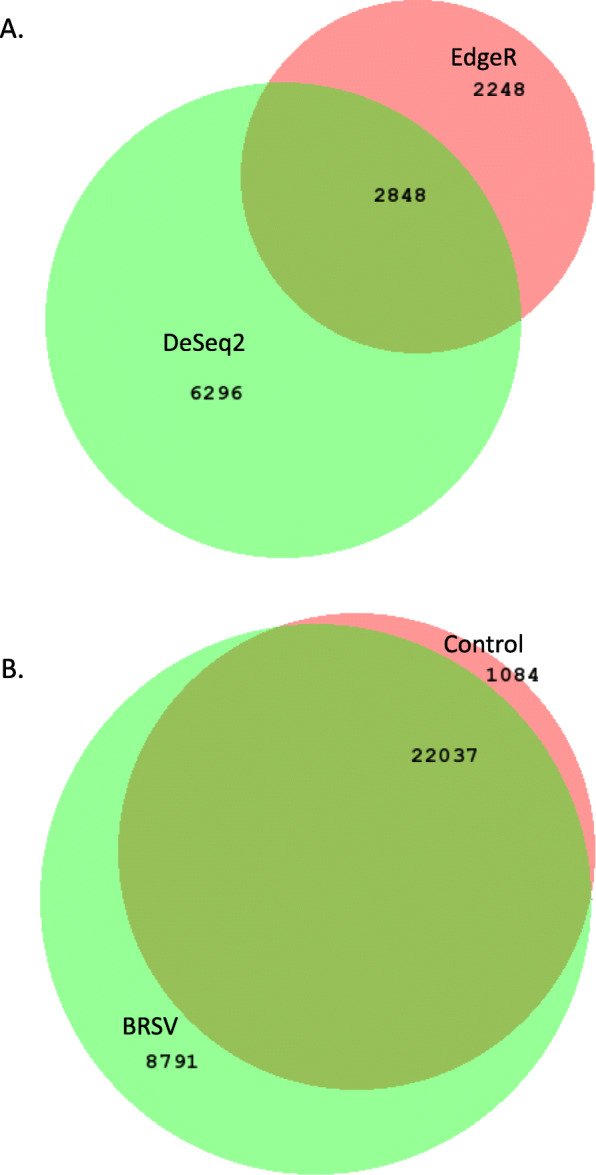
Venn diagrams showing the Diffbind accessibility and occupancy analysis results. Venn diagrams showing: **a** the number of ROCs which were predicted to be differentially accessible by DeSeq2 and EdgeR, and **b** the number of unique ROCs in bronchial lymph node tissue samples from BRSV challenged and control calves determined by Diffbind’s occupancy analysis. The Venn diagrams were produced using BioVenn [30]

**Fig. 3:**
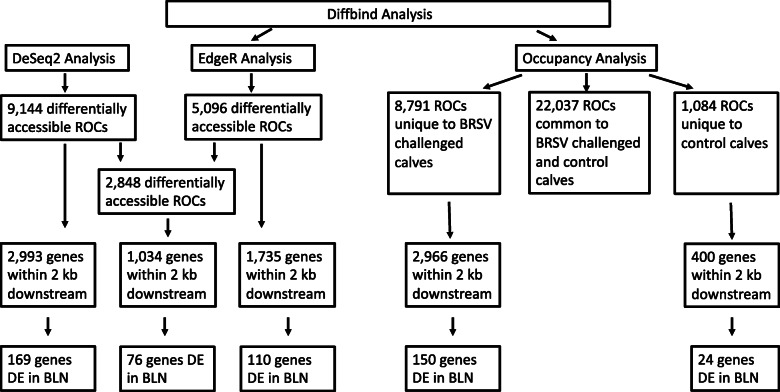
Flow chart illustrating the results of the Diffbind analysis. ROC = region of open chromatin. DE = differentially expressed. BLN = bronchial lymph node

DeSeq2 (within Diffbind) identified 9144 differentially accessible ROCs between the BRSV challenged and control calves (Additional file 5), while EdgeR identified 5096 differentially accessible ROCs (Additional file 6). There were 2848 differentially accessible ROCs found by both DeSeq2 and EdgeR (Fig. 2). There were 2993, 1735 and 1034 genes located in or within 2 kb downstream of the ROCs predicted to be differentially accessible by the DeSeq2, EdgeR and both analyses, respectively (Fig. 3). There were 169, 110 and 76 genes located in or within 2 kb downstream of the differentially accessible ROCs predicted in the DeSeq2, EdgeR and both analyses, respectively, and that were also found to be differentially expressed in the BLN RNA-Seq analysis [22] (Fig. 3). The gene set (1034 genes located in or within 2 kb upstream of the ROCs predicted to be differentially accessible by both the DeSeq2 and the EdgeR analyses) and the gene set (76 genes differentially expressed in the BLN, which were located in or within 2 kb upstream of the differentially accessible ROCs predicted to be differentially accessible by both the DeSeq2 and the EdgeR analyses) served as input data for subsequent pathway and gene ontology (GO) analyses.

Diffbind’s occupancy analysis identified 22,037, 8791 and 1084 ROCs common to both BRSV challenged and control calves, unique to BRSV challenged calves (Additional file 7) and unique to control calves (Fig. 3, Additional file 8), respectively (Fig. 2). There were 2966 and 400 genes located in or within 2 kb downstream of the ROCs which were unique to BRSV challenged calves and unique to control calves, respectively (Fig. 3). There were 150 and 24 genes located in or within 2 kb downstream of the ROCs which were unique to BRSV challenged calves and unique to control calves, respectively, and were also found to be differentially expressed in the BLN RNA-Seq analysis [22] (Fig. 3). These gene sets (located in or within 2 kb upstream of the ROCs which were (i) unique to BRSV challenged calves, (ii) unique to control calves, (iii) unique to the BRSV challenged calves and differentially expressed and (iv) unique to the control calves and differentially expressed) were provided as input to subsequent pathway and GO analyses.

### Pathway and gene ontology analysis

#### Differentially accessible ROCs found by both Deseq2 and EdgeR

**Fig. 4:**
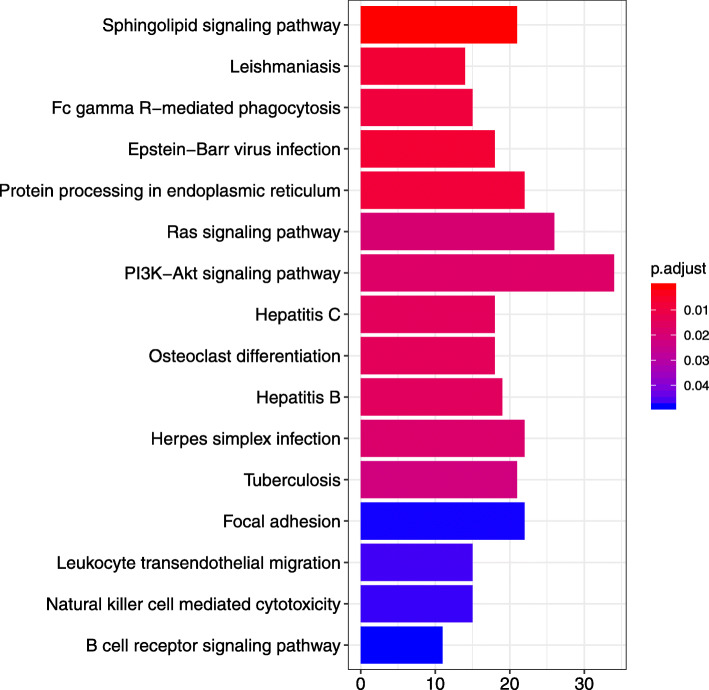
Bar chart of enriched KEGG pathways (*P* < 0.05, FDR < 0.05). Enriched KEGG pathways among the closest downstream genes to the ROCs found to be differentially accessible in bronchial lymph node tissue samples between BRSV challenged and control calves by both DeSeq2 and EdgeR. This plot was produced in ClusterProfiler based on the results of the “EnrichDAVID” function. The y-axis contains the pathway names and the x-axis defines the number of genes in each pathway which were downstream of a ROC. p.adjust = the Benjamini-Hochberg adjusted *P*-value for the enriched ontology term

**Fig. 5:**
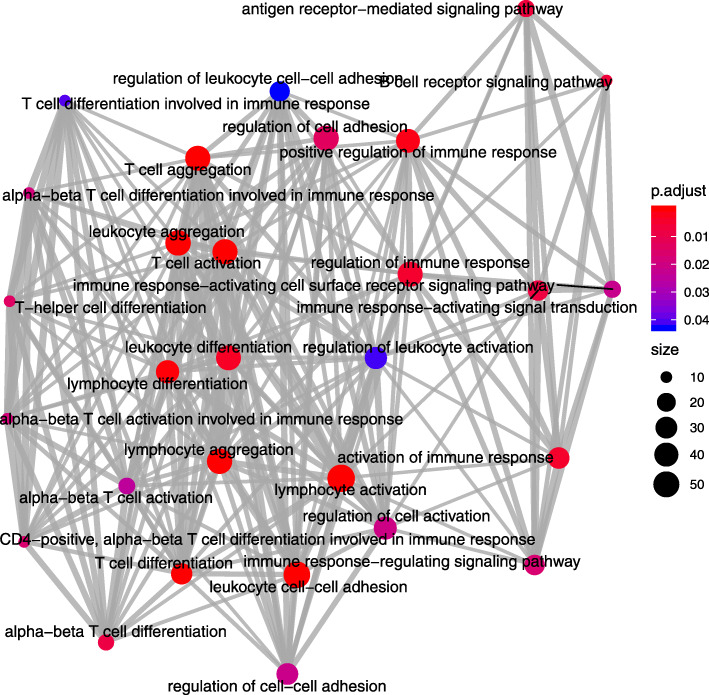
Emap plot of enriched “Biological Process” gene ontology terms (*P* < 0.05, FDR < 0.05). Enriched “Biological Process” gene ontology terms among the closest downstream genes to the ROCs predicted to be differentially accessible in bronchial lymph node tissue samples between BRSV challenged and control calves by both DeSeq2 and EdgeR. This plot was produced in ClusterProfiler based on the results of the “EnrichDAVID” function. p.adjust = the Benjamini-Hochberg adjusted P-value for the enriched ontology term. Size = the number of closest genes downstream to the differentially accessible region which belong to the enriched gene ontology term

There were 16 enriched KEGG pathways among the closest downstream genes to the differentially accessible ROCs found in both the DeSeq2 and EdgeR analyses (Fig. 4, Additional file 9). There were 29 enriched GO biological process (BP) terms (Fig. 5), 8 enriched GO molecular function (MF) and 11 enriched GO cellular component (CC) terms in the annotations for the closest downstream genes to the differentially accessible ROCs found in both the DeSeq2 and EdgeR analyses (Additional file 9).

**Fig. 6:**
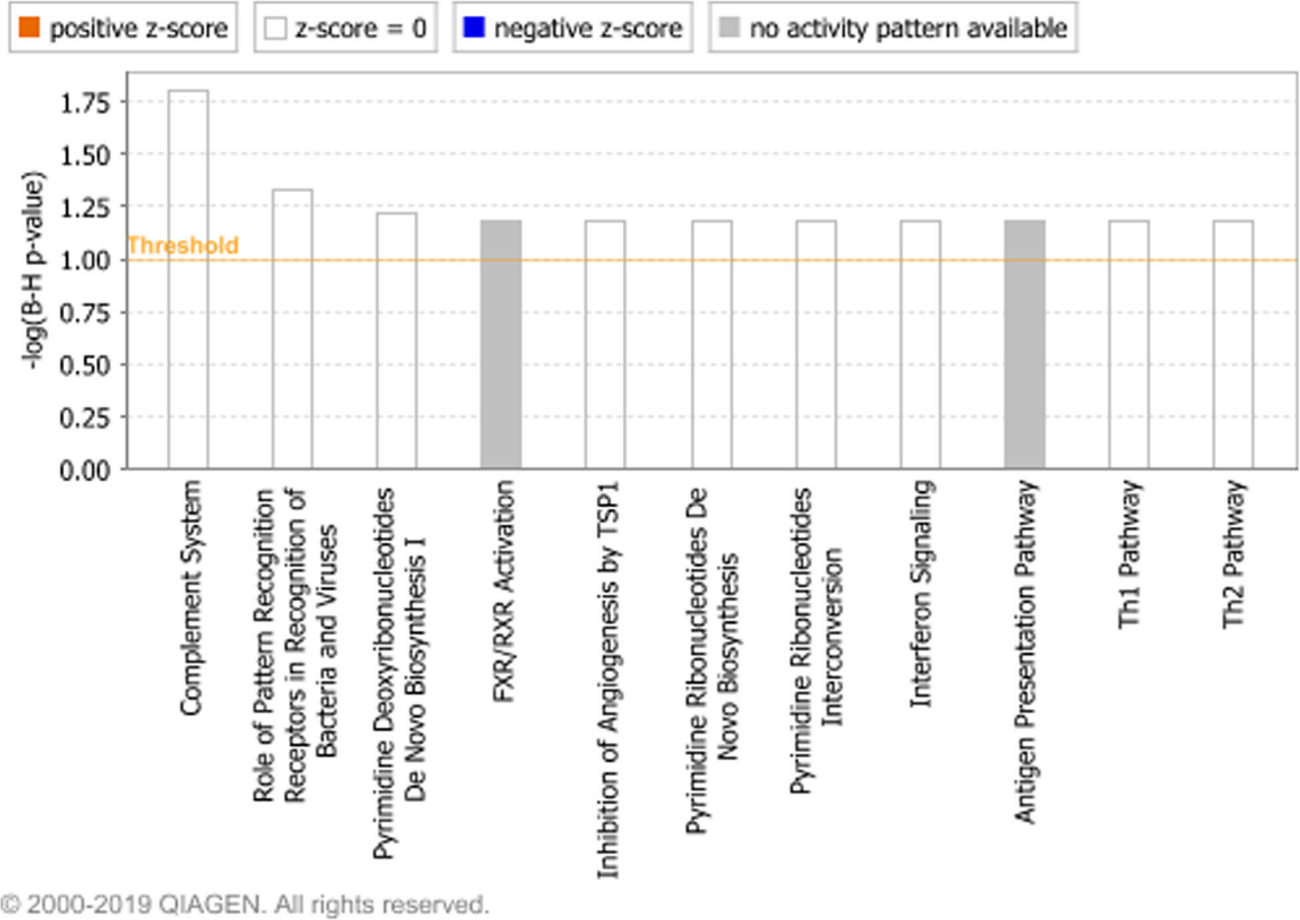
Enriched Ingenuity Pathway Analysis canonical pathways (*P* < 0.05, FDR < 0.1). Enriched pathways among the closest downstream genes to the ROCs predicted to be differentially accessible by both DeSeq2 and EdgeR, which were also predicted to be differentially expressed in the RNA-Seq analysis (*P* < 0.05, FDR < 0.1, fold change > 2). The pathways are shown on the x-axis and the –Log_10_ Benjamini-Hochberg adjusted *p*-values are displayed on the y-axis. The threshold is set to 1 which equals a Benjamini-Hochberg adjusted p-value of 0.1. Bars representing pathways with a positive, zero or negative z-score are coloured with orange, white or blue, respectively. Bars representing pathways where no activity could be determined from the z-score are coloured in grey

Differentially expressed genes and their associated fold changes, *P*-values and FDR-values from the BLN RNA-Seq study [22] which were within 2 kb downstream of a differentially accessible ROC were input to Ingenuity Pathway Analysis (IPA) which identified 11 enriched pathways (Fig. 6). One enriched IPA function was predicted to be decreased (Replication of Herpesviridae) while two enriched IPA disease and molecular functions were predicted to be increased (Cellular homeostasis and Immune response of cells). DAVID enrichment analyses performed within ClusterProfiler indicated that innate immune response, an immune related GO BP term, was enriched.

#### ROCs unique to BRSV challenged calves

There were 91 enriched KEGG pathways among the closest downstream genes to the ROCs revealed by the Diffbind occupancy analysis to be uniquely open in the BRSV challenged calves (Additional file 10). There were 187 enriched GO BP terms, 20 enriched GO MF and 41 enriched GO CC terms among the closest downstream genes to the ROCs shown by the Diffbind occupancy analysis to be uniquely open in the BRSV challenged calves (Additional file 10).

Differentially expressed genes (BRSV challenged vs. Control; *P* < 0.05, FDR < 0.1, FC > 2) within 2 kb downstream of a ROC unique to the BRSV challenged calves, and their associated fold changes, *P*-values and FDR-values from our RNA-Seq study [22], were input to IPA. Three enriched IPA molecular functions were predicted to be decreased, “neoplasia of cells”, “quantity of metal” and “incidence of tumor” and one enriched IPA molecular function was predicted to be increased “metabolism of nucleic acid component or derivative”.

#### ROCs unique to control calves

No enriched KEGG pathways were found among the closest downstream genes to the ROCs unique to the control calves identified by the Diffbind occupancy analysis. There were two enriched GO BP terms, “response to wounding” and “regulation of protein catabolic process”, and there were three enriched GO CC terms, “cell-substrate adherens”, “cell-substrate” and “focal adhesion”, among the closest downstream genes to the ROCs shown to be uniquely open in the control calves by the Diffbind occupancy analysis.

Genes within 2 kb downstream of a ROC uniquely found in the control calves which were also differentially expressed (both up- and down-regulated) in the bronchial lymph node, and their associated fold changes, P-values and FDR-values from our BLN RNA-Seq study [22], were input into IPA. There were two enriched IPA pathways; “Superpathway of Serine and Glycine Biosynthesis I” and “Serine Biosynthesis”. There were no enriched IPA diseases and molecular functions that were predicted to be either increased or decreased.

### Differentially accessible ROCs within BRD susceptibility loci

There were 237 differentially accessible ROCs identified by either DeSeq2 or EdgeR within 40 of the BRD susceptibility loci identified by Neibergs et al. [18] (Additional file 11). ROCs were identified upstream of, or within, positional candidate genes: *RDH14, BAALC, AZIN1, MAML2* and *DST* (Additional file 11). Sixteen differentially accessible ROCs were located within 7 BRD risk QTLs found in Israeli Holstein male calves by Lipkin et al. [25], 15 differentially accessible ROCs were within 7 chromosomal regions explaining the largest variance in BRD phenotypes of 3 week old calves identified by Quick et al. [20], 18 differentially accessible ROCs were within 4 large-effect BRD QTLs found in 6 week old calves by Quick et al. [20], and 1 differentially accessible ROC spanned SNP *rs29022960* which was suggestively associated with serum Immunoglobulin G concentration in Irish dairy calves [27] (Additional file 11).

There were 206 ROCs uniquely present in BRSV-challenged calves located within 42 BRD susceptibility loci identified by Neibergs et al. [18] (Additional file 12). Furthermore, there were 8 uniquely accessible ROCs detected in the BRSV-challenged calves by the Diffbind occupancy analysis located within 5 BRD QTLs identified in Israeli Holstein male calves by Lipkin et al. [25], 11 uniquely accessible ROCs identified in BRSV-challenged calves located within 5 chromosomal regions explaining the greatest variance in BRD phenotypes in 3 week old calves by Quick et al. [20], and 20 ROCs unique to BRSV-challenged calves located within 4 QTLs explaining the greatest variance in BRD phenotypes in 6 week old calves identified by Quick et al. [20] (Additional file 12).

## Discussion

To our knowledge, this is the first study to examine open chromatin regions in fresh bovine tissue samples, using ATAC-Seq, and has provided a reference resource of open chromatin regions in healthy and BRSV-challenged Holstein-Friesian calves. Chromatin is open during active gene transcription and for the regulation of transcription, as transcription factors can only be recruited to enhancers, upstream activator sequences, and proximal promoter elements of open chromatin [31]. Transcription factors subsequently recruit RNA polymerase to the core promoter for the initiation of mRNA transcription [31]. ATAC-Seq is a relatively novel, rapid, low cell input technique for the global identification of regions of open, accessible chromatin. It uses a hyperactive Tn5 transposase to insert adapter sequences into accessible chromatin regions, which can then be sequenced [28]. Omni-ATAC-Seq is a modified ATAC-Seq protocol that can be performed on frozen and fresh tissues and utilises an additional detergent step to reduce the transposition of mitochondrial derived sequences [32]. This is particularly advantageous as mitochondrial contamination is reduced and while fresh BLN tissue was utilised in this study, often it is not feasible to perform library preparation on fresh tissue immediately following collection due to a lack of available time, laboratory space, equipment or trained technicians. Furthermore, the Omni-ATAC-Seq protocol can be performed on well characterised, frozen archived tissues, to produce novel epigenetic insights [32]. The Omni-ATAC-Seq protocol was performed here to elucidate the ROCs in the BLN tissue of healthy (control) and BRSV-challenged Holstein-Friesian calves. Changes in chromatin states in response to disease status provide an insight into the regulation of the host’s transcriptional response to infection [33] and the corresponding epigenetic modifications directly induced by the pathogen [34].

ATAC-Seq has previously been performed on bovine rumen primary epithelial cells to discover changes in chromatin states induced by butyrate treatment [35], on bovine oocytes and early embryos to determine accessible chromatin regions [36], and on sorted bovine CD4+ and CD8+ primary T cells to profile accessible chromatin and identify conserved areas of open chromatin between ruminant, monogastric and bird species [37]. This study has added to the bovine chromatin accessibility knowledgebase by providing a synopsis of open chromatin regions in fresh BLN bulk tissue from 5 month old healthy dairy calves and from 5 month old dairy calves responding to an experimental challenge infection with BRSV. However, as this study utilised a specific domestic dairy breed, Holstein-Friesian, the BLN ROCs discovered may be specific to this breed type.

The majority of the ROCs found in the BLN of these dairy calves were located in protein coding genes which had previously been found to be expressed in the BLN tissue of these calves [22]. This was expected as actively transcribed genes must be at least partially accessible to RNA polymerase. Jaccard scores showed a reasonable degree of similarity between ROCs in pairwise sample comparisons; ROCs found in samples from control calves utilised in this study were 54% similar and those found in BRSV challenged calves were 59% similar to each other. Jaccard scores representing the similarity between samples within a treatment are not widely reported. However, similar Jaccard scores have been observed for stem cells and sorted blood lineage cells (0.45 to 0.60) and lower Jaccard scores for lung tumour tissues (0.10 to 0.30) [38]. As the ROCs were ascertained from DNA extracts from BLN bulk tissue samples, which contain a heterogenic mix of cell types, and because there is biological and technical variation among the analysed samples, we expected variation among samples, and a more uniform suite of ROCs would likely be generated using single cell ATAC-Seq. However, there are no published data describing the transcriptional response of individual cell types to BRSV, while there are studies describing changes in gene expression in bulk tissues [22–24], such as lung and lymph node tissues, including our RNA-Seq study [22] which elucidated the alterations in global gene expression induced in the same BLN tissue, from the same calves, and by the same BRSV challenge, as described in the present study. Therefore, it was necessary to perform the ATAC-Seq on bulk BLN tissue in order to be able to compare the ROCs with the differentially expressed genes resulting from the RNA-Seq. Samples separated out on PC2 of a PCA plot according to treatment (BRSV challenged vs. Control). There were more ROCs in the BLN from BRSV challenged calves than the control calves. This suggests that significant chromatin remodelling occurs in the BLN in response to BRSV challenge to enable the transcriptional activation of anti-viral and inflammation response genes, as the BLN is the site of antigen presentation and activation of Th1 and Th2 immune effector cells [39].

The PC1 separation on the PCA plot was associated with a combination of metrics determining library quality, including the percentage of reads which were properly paired and uniquely aligned, the percentage of reads with a MAPQ score less than 10, the percentage of mitochondrial reads and the quantity of library produced, despite an identical library preparation protocol being performed for all samples. This indicates that ATAC-Seq library preparation introduces technical variation and there may also be biological variation among individuals suggesting that sufficient biological and perhaps also technical replicates may needed to enable the identification of differences in ROCs induced due to treatment.

The enriched pathways and GO terms associated with the closest downstream genes to the differentially accessible ROCs and the ROCs that were uniquely accessible in the BRSV challenged calves are primarily involved in the immune response, particularly the anti-viral response. This is important as it shows that the differentially and uniquely accessible regions of chromatin in the BLN of the BRSV challenged calves function as expected, in the immune-based transcriptional response to the virus. Enriched KEGG pathways including specific viral pathways (“Epstein-Barr virus infection”, “Influenza A”, “Hepatitis C”, “Hepatitis B”, “Herpes simplex infection”) and pathways related to pathogen recognition, cytotoxic and phagocytic cells “Fc gamma R-mediated phagocytosis”, “Natural killer cell mediated cytotoxicity”, “chemokine signalling pathway”, “Toll-like receptor signalling pathway”, “TNF signalling pathway”, “NF-kappa B signalling pathway” and “Leukocyte transendothelial migration”, further emphasize the increased transcription of genes involved in the innate and anti-viral immune responses. Similarly, the enriched GO terms associated with these genes including “T cell activation”, “T cell differentiation”, “T cell aggregation”, “T cell mediated immunity”, “interferon-gamma production”, “antigen receptor-mediated signalling pathway”, “immune response-activating signal transduction” and “T-helper cell differentiation”, demonstrate the inflammatory T-cell response in the BLN to BRSV challenge [39]. However, B cell receptor signalling was also an enriched KEGG pathway and GO terms associated with the antibody mediated B cell response were enriched, which is consistent with the notion that BRSV can bias the host’s acquired immune response towards a less efficient Th2 response [40].

There were enriched GO terms associated with the closest downstream genes of ROCs uniquely present in the BRSV-challenged calves that were related to neurotransmitter synapses, including dopaminergic synapse, serotonergic synapse, GABAergic synapse, glutamatergic synapse and cholinergic synapse. Lymphatic vessels contract and dilate in order to transport fluid, lipids and immune cells, and their contractibility is vital for the maintenance of homeostasis [41]. This suggests that infection with BRSV may affect the regulation of lymph vessel contractibility and/or vasodilation and may consequently affect the trafficking of immune effector cells to the site of infection. However, neurotransmitter synapses innervating lymphatic vessels need to be identified by immunohistochemistry or a similar technique with the capability of resolving molecular levels spatially.

Integrating the ATAC-seq detected ROCs with the differentially expressed genes from the BLN RNA-Seq study [22] revealed the dependence between differentially expressed genes and differentially accessible or uniquely accessible ROCs in the BRSV challenged relative to control calves. Differentially expressed genes involved in the innate immune response, the complement system, antigen presentation, the Th1 and Th2 signalling pathways, interferon signalling and pathogen recognition all had a differentially accessible ROC located either within, or within 2 kb upstream, of the gene. This confirms the positive relationship between regions of open chromatin and increased levels gene expression.

The identified ROCs which are differentially or uniquely open in the BRSV challenged calves are involved in the transcriptional and regulatory response (including transcription factor binding, DNA methylation, mRNA splicing and mRNA stability) to infection with BRSV. As complex traits, including BRD risk, are controlled by multiple small effect genetic variants and environmental factors, it is very difficult to identify causative disease associated variants [42]. However, as the differentially and uniquely accessible ROCs in the BLN in response to BRSV challenge are involved in the regulation of immune responses, they may be interrogated in future large scale or functional studies for the identification of variants associated with resistance to BRSV infection. There were several differentially accessible and uniquely accessible ROCs in the BLN of BRSV-challenged calves that were located in BRD susceptibility loci previously identified by GWAS [18, 20, 25]. These regions are likely to harbour causative genetic variants for BRD susceptibility, particularly to BRSV. These differentially open regions include positional candidate genes [18] including *RDH14, BAALC, AZIN1, MAML2* and *DST* as their closest downstream genes. These positional candidate genes are associated with various diseases or immune related functions. *RDH14* is involved in retinol metabolism [43], increased expression of *BAALC* has been implicated in myeloid leukemogenesis [44], mutations in *AZIN1* are associated with the progression of liver fibrosis during hepatitis C infection [45], *DST* is involved in the transport of herpes simplex virus 1 capsids to the nucleus of the host cell [46] and *MAML2* is associated with risk of mucoepidermoid carcinoma [47].

As 90% of variants identified by GWAS studies are located in non-protein-coding regions of the genome [42], elucidating the variants associated with BRD resistance which are located in differentially or uniquely open ROCs in the BRSV challenged calves could identify their roles in the regulation of gene expression in immune and anti-viral responses and could lead to improved disease resistance through selection on these variants. The *rs29022960* SNP in an intron of *ZNF292* was previously observed to be suggestively associated with serum Immunoglobulin G concentration, a measure of passive immune status, in Irish dairy calves [27] and is located in a differentially accessible ROC. As calves with a superior passive-derived immunity are less likely to develop respiratory diseases [6], this SNP may be influencing gene regulatory events which impact immunity and the ability to resist BRD. The identification of causal BRD risk variants will enable more accurate and precise estimates of genetic merit for disease resistance across breeds of cattle than the common marker variants currently used in GWAS and genomic selection that are in linkage disequilibrium with causative mutations [18]. Their identification would allow their inclusion in the genotyping assays routinely used by the international beef and dairy cattle industries for the generation of healthier, more robust cattle with a greater potential to resist BRSV infection and subsequent BRD.

## Conclusions

This is the first study to use ATAC-Seq to eluicidate ROCs in fresh bovine tissue samples. The study has provided the bovine genomics research community with a comprehensive resource of ROCs identified in healthy and BRSV-challenged Holstein-Friesian calves. Differentially and uniquely accessible regions of chromatin found in calves experimentally challenged with BRSV are located close to genes involved in the immune response, particularly the anti-viral response. Several BRD risk QTLs previously identified by GWAS are located within differentially and uniquely accessible ROCs found in this study. These ROCs may harbour genetic variants that create biological variation in the transcriptional and regulatory response to infection by BRD. Further interrogation of the variants in these regions for their association with risk of BRD will identify markers that will be broadly useful across breeds for use in breeding programmes to improve the genetic merit of health traits in beef and dairy cattle.

## Methods

### Animal model

The animal model has been published in Johnston et al. [22]. Briefly, clinically healthy Holstein-Friesian calves (Mean age 143 ± 14 days, Mean weight 155 ± 14 kg) were either challenged with BRSV inoculum (10^3.5^ TCID_50_/ml × 15 ml) (*n* = 12) (BRSV challenged) or mock challenged with phosphate buffered saline (*n* = 6) (Control) by aerosol inhalation, at the Agri-Food Biosciences Institute (AFBI), Stormont, Belfast, UK. Clinical assessments were performed daily. On day 7 relative to the challenge, calves were euthanised by captive bolt, the lungs were removed, assessed and scored by a veterinary practitioner and respiratory associated tissues (bronchial, retropharyngeal and mediastinal lymph nodes, pharyngeal tonsil) were collected. The workspace and instruments were thoroughly cleaned and disinfected with bleach, 75% ethanol and RNaseZap, before tissue collection and between euthanisation of animals. Bronchial lymph node tissues were harvested immediately and transported on ice to the laboratory for the first stage of ATAC-Seq library preparation (cell lysis, nuclei isolation and transposition).

### ATAC-Seq library preparation

ATAC-Seq libraries were prepared from 2 mg of fresh BLN tissue according to the Corces et al. [32] “Omni-ATAC” protocol with a modification of the transposase enzyme quantity from 2.5 μl to 5 μl. Bronchial lymph node tissue was lysed using a tissue dounce, and nuclei were obtained by density centrifugation. Nuclei were counted with a light microscope and 50,000 nuclei were transposed for 30 min, at 37 °C in a thermomixer (1000 RPM). The Tn5 transposase enzyme (5 μl) and the TD reaction buffer 25 μl from the Illumina Nextera DNA library preparation kit (cat no. FC-121-1030) (Illumina, Inc., San Diego, California) were used in the transposition reaction. The transposed DNA was purified using the Zymo DNA Clean and Concentrator-5 kit (Cambridge Bioscience, United Kingdom). The libraries were frozen at − 80 °C and transported on dry ice to Teagasc Grange, Dunsany, Ireland where the second stage of ATAC-Seq library preparation (PCR amplification) was performed.

Libraries were initially PCR amplified in an Eppendorf 5331 Mastercycler Gradient v2.30.31 thermocycler for 5 cycles to incorporate the Illumina Nextera i5 and i7 indexes from the Nextera Index Kit (cat no. FC-121-1011). Real time qPCR was carried out on 5 μl of each library using the Applied Biosystems 7500 FAST RT-PCR equipment v2.0.1 (Applied Biosystems, California, USA) to determine the optimal number of additional PCR cycles to be performed on each of the libraries. The number of additional PCR cycles was determined by plotting the linear reaction versus cycle and determining the cycle number which corresponded to one third of the maximum florescence intensity [32]. The libraries were each PCR amplified for the appropriate number of additional cycles and were then purified with the Zymo DNA Clean and Concentrator-5 kit (Cambridge Bioscience, United Kingdom).

The quality of the ATAC-Seq libraries was examined using an Agilent 2100 Bioanalyser (Agilent Technologies Ireland Ltd.; Dublin, Ireland) with an Agilent High Sensitivity DNA kit (Agilent Technologies Ireland Ltd.; Dublin, Ireland). Libraries were quantified with a Qubit Fluorometer.

### Sequencing of ATAC-Seq libraries

ATAC-Seq libraries were shipped frozen at − 80 °C on dry ice to the University of Missouri’s DNA Core Facility for high-throughput sequencing (75 bp paired-end) on an Illumina NextSeq 500.

### Bioinformatics and data analysis of ATAC-Seq libraries

Sequence reads (FASTQ format) were examined for quality using FastQC (version 0.11.8) (http://www.bioinformatics.babraham.ac.uk/projects/fastqc/) and 3′ trimmed for Nextera adapters, low quality reads (quality score < 20), ambiguous nucleotides, and poly-G artefacts resulting from the NextSeq’s two-colour chemistry, using CutAdapt (version 1.18). Trimmed reads were quality assessed with FastQC and all reads passed the basic quality statistics, following adapter trimming. Reads were aligned to the UMD3.1 bovine reference genome assembly using Bowtie2 (version 2.3.4) [48] and alignments were output as bam files. Fragment size distribution graphs were created with Picard (version 2.18.23–0) (http://broadinstitute.github.io/picard/) (Additional file 13). Mitochondrial reads, reads with a MAPQ < 10 and reads which did not align to the reference genome were removed using Samtools (version 1.9) [49]. Picard (version 2.18.23–0) mark duplicates was used to determine the number of duplicate sequence reads. Peak calling (q < 0.01) was performed with MACS2 (version 2.1.4) [50], using the BAMPE model and removing all duplicate reads.

Bedtools (version 2.27.1) [51] Jaccard was used for pairwise comparisons of all samples to determine the number of peak intersections and to score the similarity between samples.

Diffbind (version 2.14.0) [52, 53] was used to identify differentially accessible regions (peaks). Peaksets from MACS2, and associated metadata, for each sample were read into Diffbind within R (version 3.6.1 (2019-07-05) -- “Action of the Toes”). A single set of unique genomic intervals (referred to here as ROCs) covering all the peaks within all samples (a consensus peakset) was derived from all overlapping peaks. The number of reads which overlapped each consensus peak interval (ROC) for each sample was quantified. DeSeq2 [54] and EdgeR [55] were employed within Diffbind for the identification of ROCs which were differentially accessible between the BRSV challenged and control calves (*P* < 0.05, FDR < 0.05). Additionally, ROCs unique to either BRSV challenged calves or control calves were determined using Diffbind’s occupancy analysis. Consensus ROCs for BRSV challenged calves and for control calves were generated with the requirement that peaks overlapped in at least 66% of the samples within each of the BRSV challenged or control sample groups. The ROCs which were unique to the BRSV challenged calves and to the control calves were identified. Bedtools (version 2.27.1) closest was used to determine the closest genes downstream of the identified ROCs.

### Pathway and gene ontology analysis

Three gene lists were created with each containing the closest genes within 2 kb downstream of the ATAC-Seq ROCs which were: 1) differentially accessible between the BRSV challenged and control calves in both Diffbind’s EdgeR and DeSeq2 analyses, 2) uniquely present in the BRSV challenged calves, and 3) uniquely present in the control calves. These three gene lists were separately input into ClusterProfiler (version v3.14.0) [56] in R (version 3.6.1), for DAVID (version 6.8) pathway and GO analysis [57–59] using the “EnrichDAVID” function. The annotation types interrogated included: “GOTERM_BP_ALL”, “GOTERM_MF_ALL”, “GOTERM_CC_ALL” and “KEGG_PATHWAY”. Pathways and GO terms from the DAVID ClusterProfiler analyses were considered enriched at a *P*-value of less than 0.05 and an FDR of 5%.

The three gene lists were merged in R (version 3.6.1) with the genes found to be differentially expressed in the RNA-Seq dataset (*P* < 0.05, FDR < 0.1, FC > 2) [22], to obtain three new lists comprising only the genes which were differentially expressed in the BLN. These genes, along with their corresponding RNA-Seq fold changes, FDR and *P*-values were examined for over-represented pathways, cellular and molecular functions, using the Ingenuity Pathway Analysis (QIAGEN Inc., https://www.qiagenbioinformatics.com/products/ingenuitypathway-analysis), according to the manufacturer’s instructions [60]. Within IPA, Fisher’s exact test was used with the Benjamini-Hochberg correction for multiple testing for the identification of over-represented pathways and over-represented molecular and cellular functions with a FDR of 10%. Additionally, IPA’s regulation Z-score algorithm, which predicts increases or decreases in functions based on directional changes in the differentially expressed genes was used to predict differences in the over-represented cellular and molecular functions. IPA software considered cellular and molecular functions with a regulation Z-score value of ≥2.0 to be significantly increased and cellular and molecular functions with a regulation Z-score value of ≤ − 2.0 to be significantly decreased. These genes and their corresponding RNA-Seq fold changes were further analysed for enriched KEGG pathways and GO terms using the “EnrichDAVID” function in ClusterProfiler. Pathways and GO terms from the DAVID ClusterProfiler analyses were considered enriched at a P-value of less than 0.05 and an FDR of 5%.

### Comparison of differentially accessible ROCs with BRD susceptibility associated loci and genetic variants

The ROCs found to be: (i) differentially accessible by either Diffbind’s DeSeq2 or EdgeR analyses, or (ii) uniquely accessible in BRSV-challenged calves, were compared with: BRD susceptibility loci identified by Neibergs et al. [18], QTLs explaining variance in BRD phenotypes in 3 or 6 week old calves identified by Quick et al. [20], QTLs and SNPs associated with BRD risk in Israeli Holstein male calves identified by Lipkin et al. [25], SNPs associated with lung lesion severity determined by Keele et al. [26], and SNPs found to be associated with passive immune and disease traits in Irish dairy and beef calves by Johnston et al. [27]. The differentially accessible ROCs, and the ROCs that were predicted to be unique to BRSV-challenged calves, that were located within the BRD susceptibility loci or that contained a BRD associated SNP, were identified using Bedtools (version 2.27.1) intersect. The closest genes downstream of the ROCs that were either differentially accessible or unique to the BRSV-challenged calves were identified using Bedtools (version 2.27.1) closest.

## Supplementary Information

**Additional file 1.** Sequence read statistics: the total read numbers, percentages of mapped reads, mitochondrial mapped reads and duplicate reads, the non-redundant fractions and the number of reads used for peak calling.

**Additional file 2.** Matrix displaying the number of peaks per sample, the number of peak intersections between pairwise comparisons and the BEDtools Jaccard similarity score between pairwise comparisons.

**Additional file 3.** Flow chart illustrating ROCs proximity to protein-coding genes. ROC = region of open chromatin. BLN = bronchial lymph node.

**Additional file 4. **Principal component analysis plots. Principal component plots of ATAC-Seq regions of accessible chromatin (ROC) data for bronchial lymph node tissue samples from BRSV challenged and control calves. These plots were generated in Diffbind and illustrate the similarity of the BRSV challenged (*n* = 12) and control (*n* = 6 and/or *n* = 4) calves’ bronchial lymph node samples based on ATAC-Seq ROC. Bronchial lymph node tissue samples from BRSV challenged calves (Calf IDs 7 to 18) are coloured in pink and from control calves (Calf IDs 1 to 6) are coloured in purple. a) Principal component analysis (PCA) plot of all samples. b) PCA plot after removal of control samples 4 and 5. c) PCA plot with superimposed percentages of reads properly paired and uniquely aligned. d) PCA plot with superimposed percentages of mitochondrial reads per library. e) PCA plot with superimposed percentages of reads with a MAPQ score less than 10. f) PCA plot with superimposed non-redundant fractions. g) PCA plot with superimposed number of additional qPCR cycles performed during library preparation. h) PCA plot with superimposed library quantities produced (ng/μl).

**Additional file 5. **Differentially accessible regions (peaks) from the DeSeq2 analysis within the Diffbind R package, between BRSV challenged and control calves (*P* < 0.05, FDR < 0.05).

**Additional file 6. **Differentially accessible regions (peaks) from the EdgeR analysis within the Diffbind R package, between BRSV challenged and control calves (*P* < 0.05, FDR < 0.05).

**Additional file 7.** Regions (peaks) unique to BRSV challenged calves identified through Diffbind’s occupancy analysis.

**Additional file 8.** Regions (peaks) unique to the control calves identified through Diffbind’s occupancy analysis.

**Additional file 9. **Enriched KEGG pathways and “Biological process (BP)”, “Molecular Function (MF)” and “Cellular Component (CC)” Gene Ontology (GO) terms (*P* < 0.05, FDR < 0.05) among the closest downstream genes to the regions found to be differentially accessible between BRSV challenged and control calves by both DeSeq2 and EdgeR.

**Additional file 10. **Enriched KEGG pathways and “Biological process (BP)”, “Molecular Function (MF)” and “Cellular Component (CC)” Gene Ontology (GO) terms (*P* < 0.05, FDR < 0.05) among the closest downstream genes to the regions uniquely open in the BRSV challenged calves.

**Additional file 11.** The regions (peaks) which were differentially accessible in either DeSeq2 or EdgeR, which were positioned in previously identified BRD susceptibility loci and BRD associated marker variants.

**Additional file 12.** The regions (peaks) which were uniquely accessible in BRSV-challenged calves, which were positioned in previously identified BRD susceptibility loci and BRD associated marker variants.

**Additional file 13.** Fragment size distribution graphs created with Picard for the samples from the control calves (S1-S6) and the BRSV challenged calves (S7-S18). FR = forward reverse orientation. RF = reverse forward orientation.

## Data Availability

All sequence data produced in this study have been deposited to the NCBI GEO repository and are available through the series accession number GSE148056.

## Abbreviations

AFBI: Agri-Food Biosciences Institute
ATAC-Seq: Assay for Transposase-Accessible Chromatin using Sequencing
BLN: Bronchial lymph node
BP: Biological process
bp: Base pair
BRD: Bovine Respiratory Disease
BRDC: Bovine Respiratory Disease Complex
BRSV: Bovine Respiratory Syncytial Virus
CC: Cellular component
FC: Fold change
FDR: False discovery rate
GO: Gene ontology
GWAS: Genome wide association study
IPA: Ingenuity Pathway Analysis
kb: Kilobase
MF: Molecular function
PBS: Phosphate buffered saline
PCA: Principal component analysis
qPCR: Quantitative polymerase chain reaction
ROC: Region of open chromatin
QTL: Quantitative trait locus
SD: Standard deviation
SEM: Standard error of the mean
SNP: Single nucleotide polymorphism
TCID_50_: Fifty-percent tissue culture infective dose
Th: T helper
