# Supplementary material for: ATAC-Seq identifies regions of open chromatin in the bronchial lymph nodes of dairy calves experimentally challenged with bovine respiratory syncytial virus
Source: BMC Genomics. 2021 Jan 6;22:14. doi: 10.1186/s12864-020-07268-5 (PMC7789798; doi:10.1186/s12864-020-07268-5)
Supplement: Supplementary file 3 — Additional file 3. Flow chart illustrating ROCs proximity to protein-coding genes. ROC = region of open chromatin. BLN = bronchial lymph node. [file 12864_2020_7268_MOESM3_ESM.pdf]

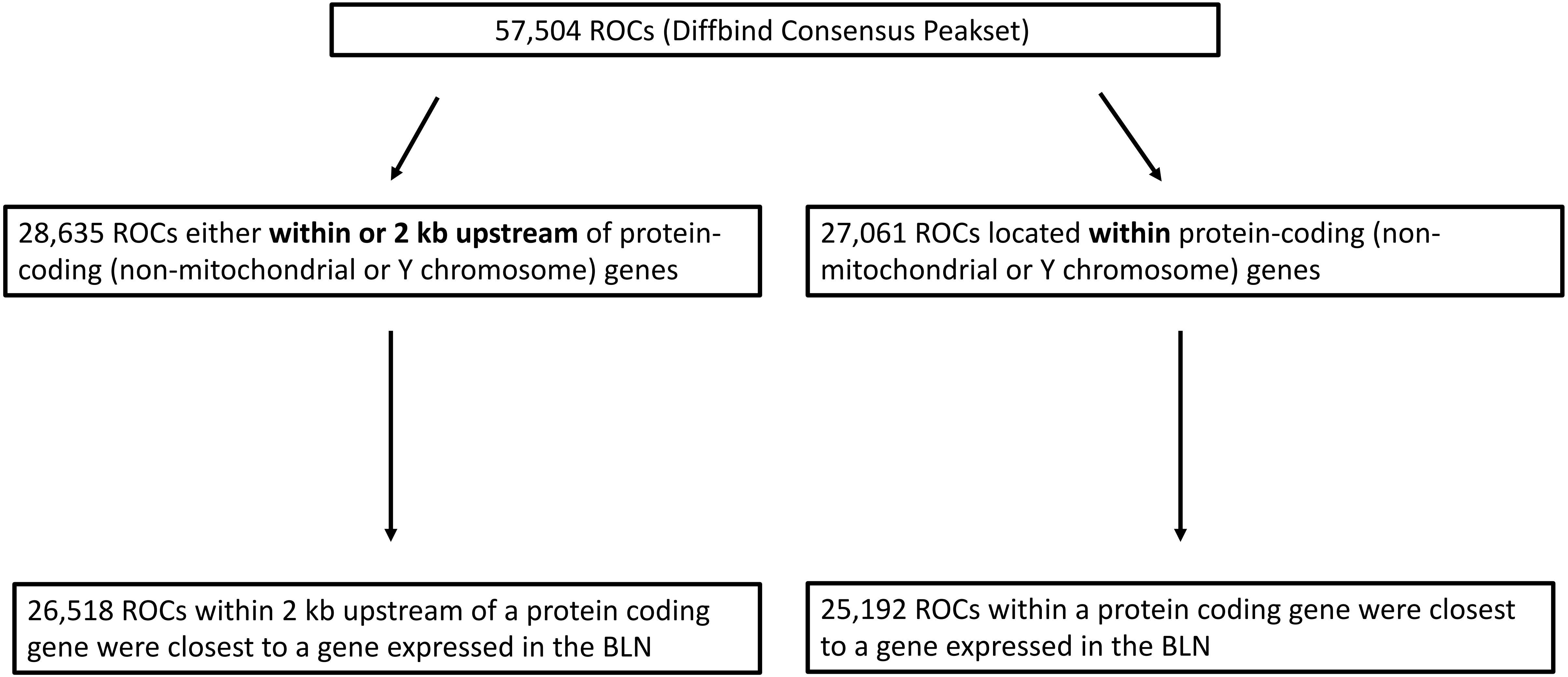

ROC = Region of open chromatin, BLN =Bronchial lymph node
