# Supplementary material for: ATAC-Seq identifies regions of open chromatin in the bronchial lymph nodes of dairy calves experimentally challenged with bovine respiratory syncytial virus
Source: BMC Genomics. 2021 Jan 6;22:14. doi: 10.1186/s12864-020-07268-5 (PMC7789798; doi:10.1186/s12864-020-07268-5)
Supplement: Supplementary file 4 — Additional file 4. Principal component analysis plots. Principal component plots of ATAC-Seq regions of accessible chromatin (ROC) data for bronchial lymph node tissue samples from BRSV challenged and control calves. These plots were generated in Diffbind and illustrate the similarity of the BRSV challenged (n = 12) and control (n = 6 and/or n = 4) calves’ bronchial lymph node samples based on ATAC-Seq ROC. Bronchial lymph node tissue samples from BRSV challenged calves (Calf IDs 7 to 18) are coloured in pink and from control calves (Calf IDs 1 to 6) are coloured in purple. a) Principal component analysis (PCA) plot of all samples. b) PCA plot after removal of control samples 4 and 5. c) PCA plot with superimposed percentages of reads properly paired and uniquely aligned. d) PCA plot with superimposed percentages of mitochondrial reads per library. e) PCA plot with superimposed percentages of reads with a MAPQ score less than 10. f) PCA plot with superimposed non-redundant fractions. g) PCA plot with superimposed number of additional qPCR cycles performed during library preparation. h) PCA plot with superimposed library quantities produced (ng/μl). [file 12864_2020_7268_MOESM4_ESM.pdf]

Additional File 4. Principal component analysis plots.

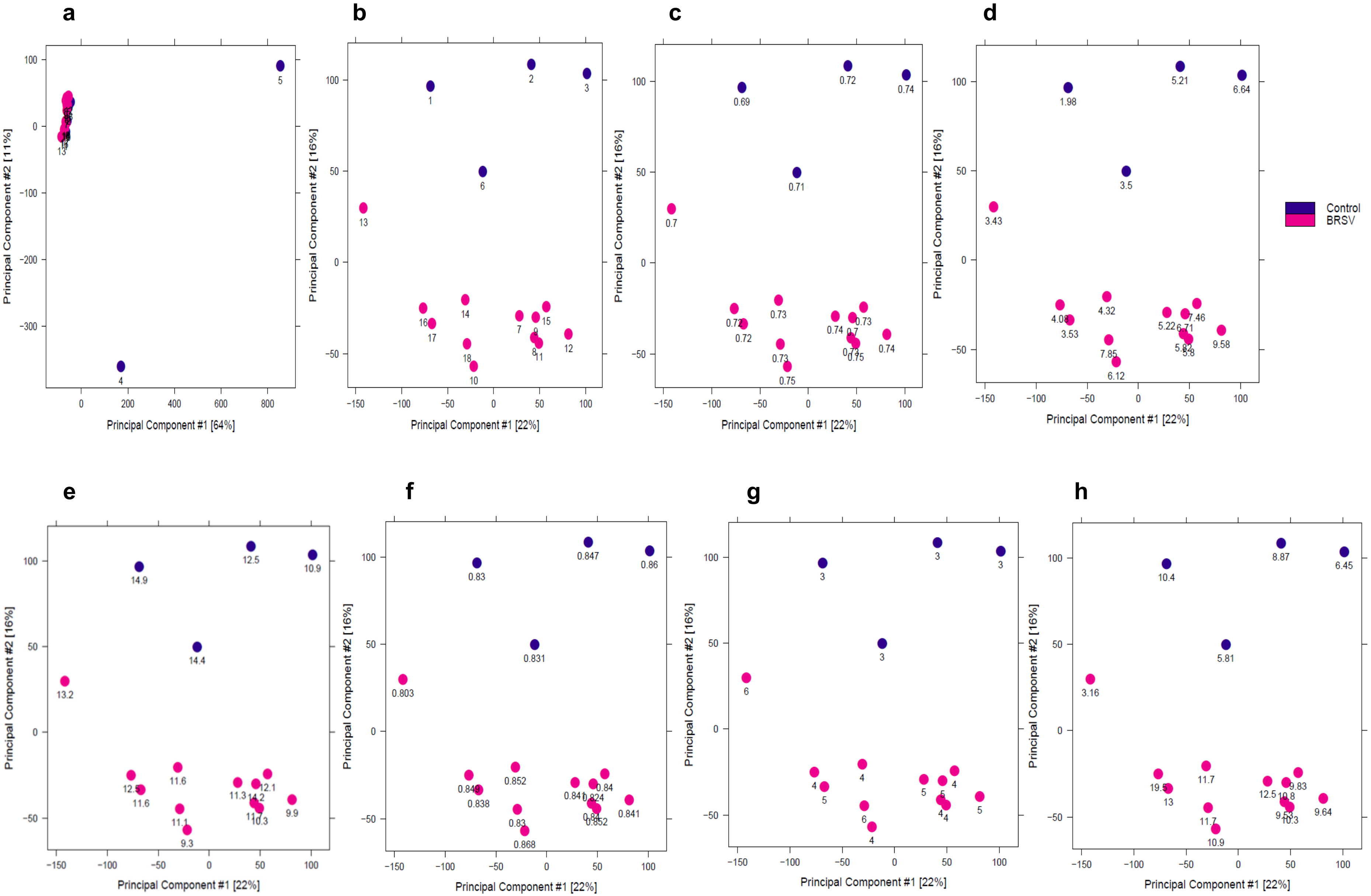

Principal component plots of ATAC-Seq regions of accessible chromatin (ROC) data for bronchial lymph node tissue samples from BRSV challenged and control calves. These plots were generated in Diffbind and illustrate the similarity of the BRSV challenged (n=12) and control (n=6 and/or n=4) calves' bronchial lymph node samples based on ATAC-Seq ROC. Bronchial lymph node tissue samples from BRSV challenged calves (Calf IDs 7 to 18) are coloured in pink and from control calves (Calf IDs 1 to 6) are coloured in purple. a) Principal component analysis (PCA) plot of all samples. b) PCA plot after removal of control samples 4 and 5. c) PCA plot with superimposed percentages of reads properly paired and uniquely aligned .d) PCA plot with superimposed percentages of mitochondrial reads per library. e) PCA plot with superimposed percentages of reads with a MAPQ score less than 10. f) PCA plot with superimposed non-redundant fractions. g) PCA plot with superimposed number of additional qPCR cycles performed during library preparation. h) PCA plot with superimposed library quantities produced (ng).
