# Supplementary material for: ATAC-Seq identifies regions of open chromatin in the bronchial lymph nodes of dairy calves experimentally challenged with bovine respiratory syncytial virus
Source: BMC Genomics. 2021 Jan 6;22:14. doi: 10.1186/s12864-020-07268-5 (PMC7789798; doi:10.1186/s12864-020-07268-5)
Supplement: Supplementary file 13 — Additional file 13. Fragment size distribution graphs created with Picard for the samples from the control calves (S1-S6) and the BRSV challenged calves (S7-S18). FR = forward reverse orientation. RF = reverse forward orientation. [file 12864_2020_7268_MOESM13_ESM.pdf]

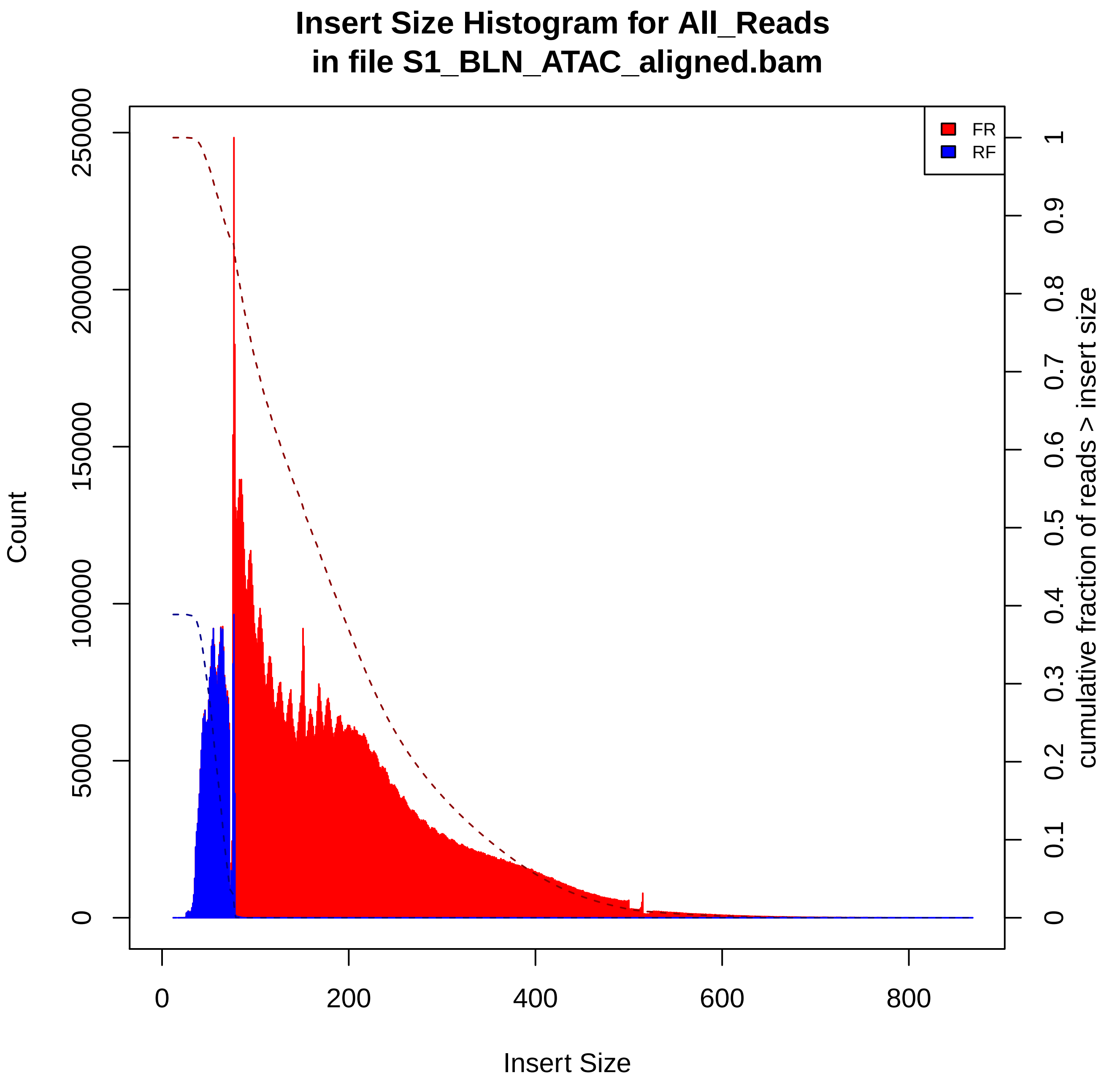
Insert Size Histogram for All_Reads
in file S1_BLN_ATAC_aligned.bam
FR
RF
Count
250000
200000
150000
100000
50000
0
cumulative fraction of reads > insert size
1
0.9
0.8
0.7
0.6
0.5
0.4
0.3
0.2
0.1
0
0
200
400
600
800
Insert Size

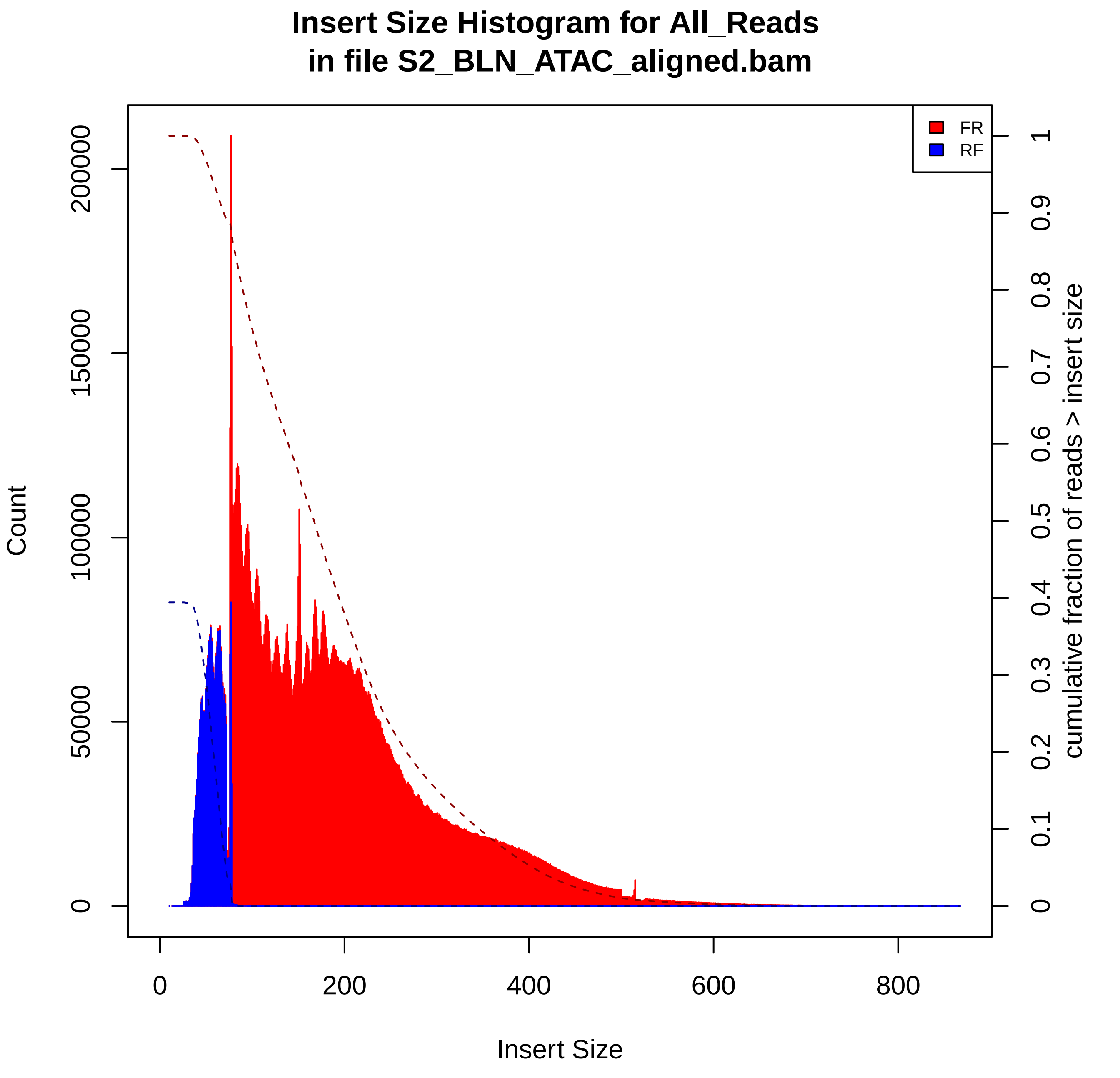
Insert Size Histogram for All_Reads
in file S2_BLN_ATAC_aligned.bam
FR
RF
Count
0
50000
100000
150000
200000
cumulative fraction of reads > insert size
0
0.1
0.2
0.3
0.4
0.5
0.6
0.7
0.8
0.9
1
0
200
400
600
800
Insert Size

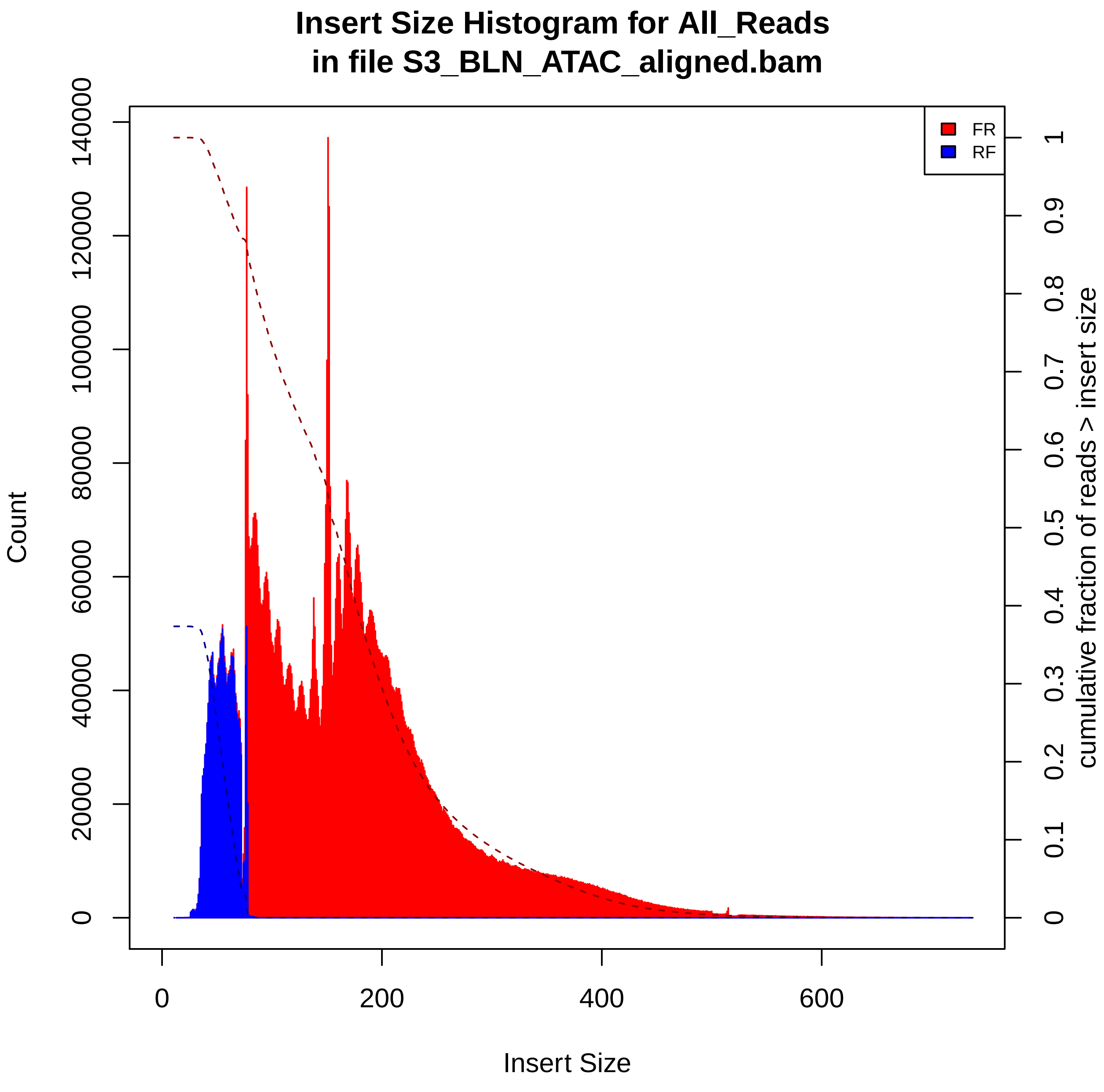

Insert Size Histogram for All_Reads
in file S3_BLN_ATAC_aligned.bam
Count
0
20000
40000
60000
80000
100000
120000
140000
FR
RF
0
200
400
600
Insert Size
cumulative fraction of reads > insert size
0
0.1
0.2
0.3
0.4
0.5
0.6
0.7
0.8
0.9
1

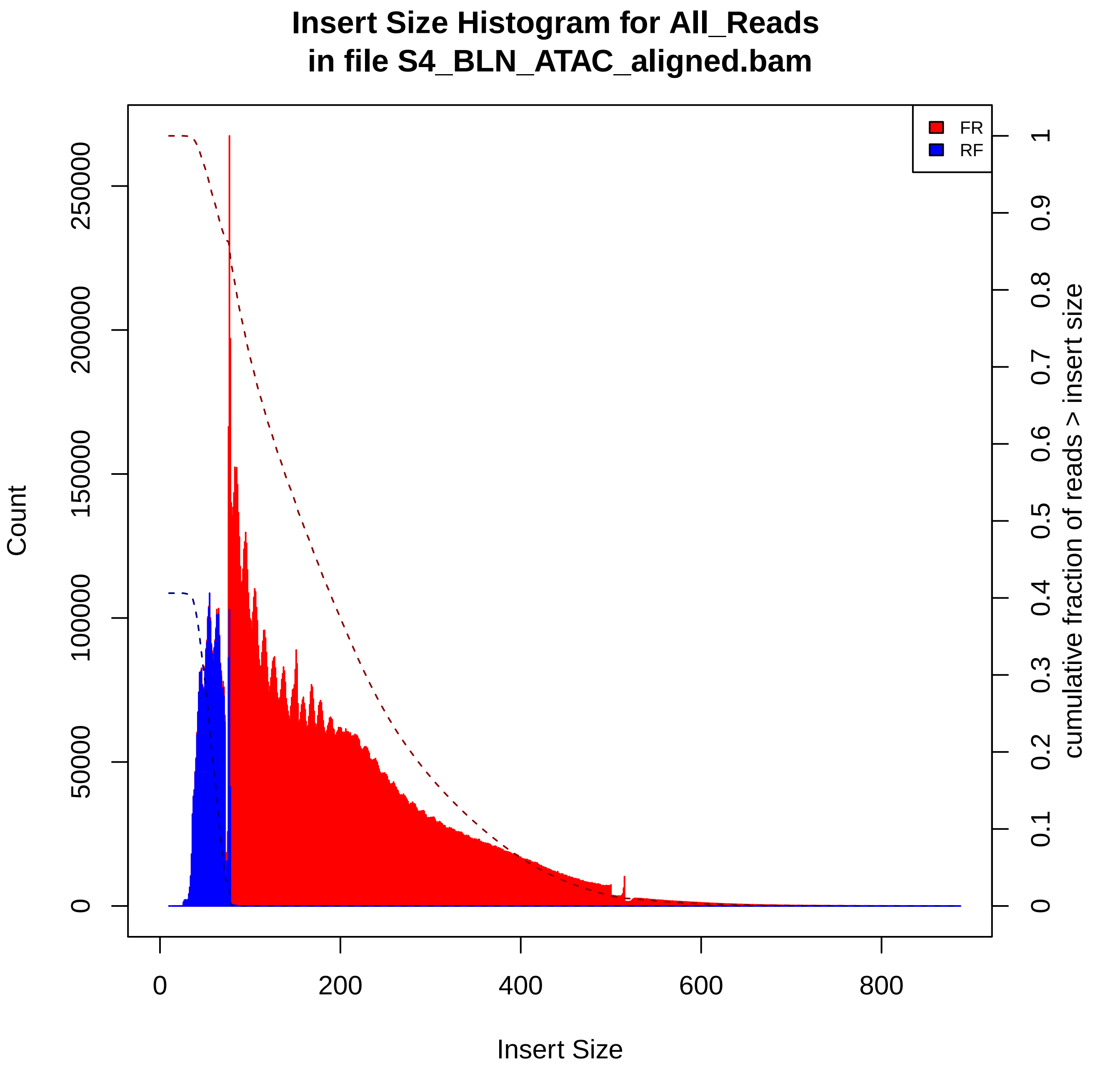

Insert Size Histogram for All_Reads
in file S4_BLN_ATAC_aligned.bam
FR
RF
Count
0
50000
100000
150000
200000
250000
cumulative fraction of reads > insert size
0
0.1
0.2
0.3
0.4
0.5
0.6
0.7
0.8
0.9
1
0
200
400
600
800
Insert Size

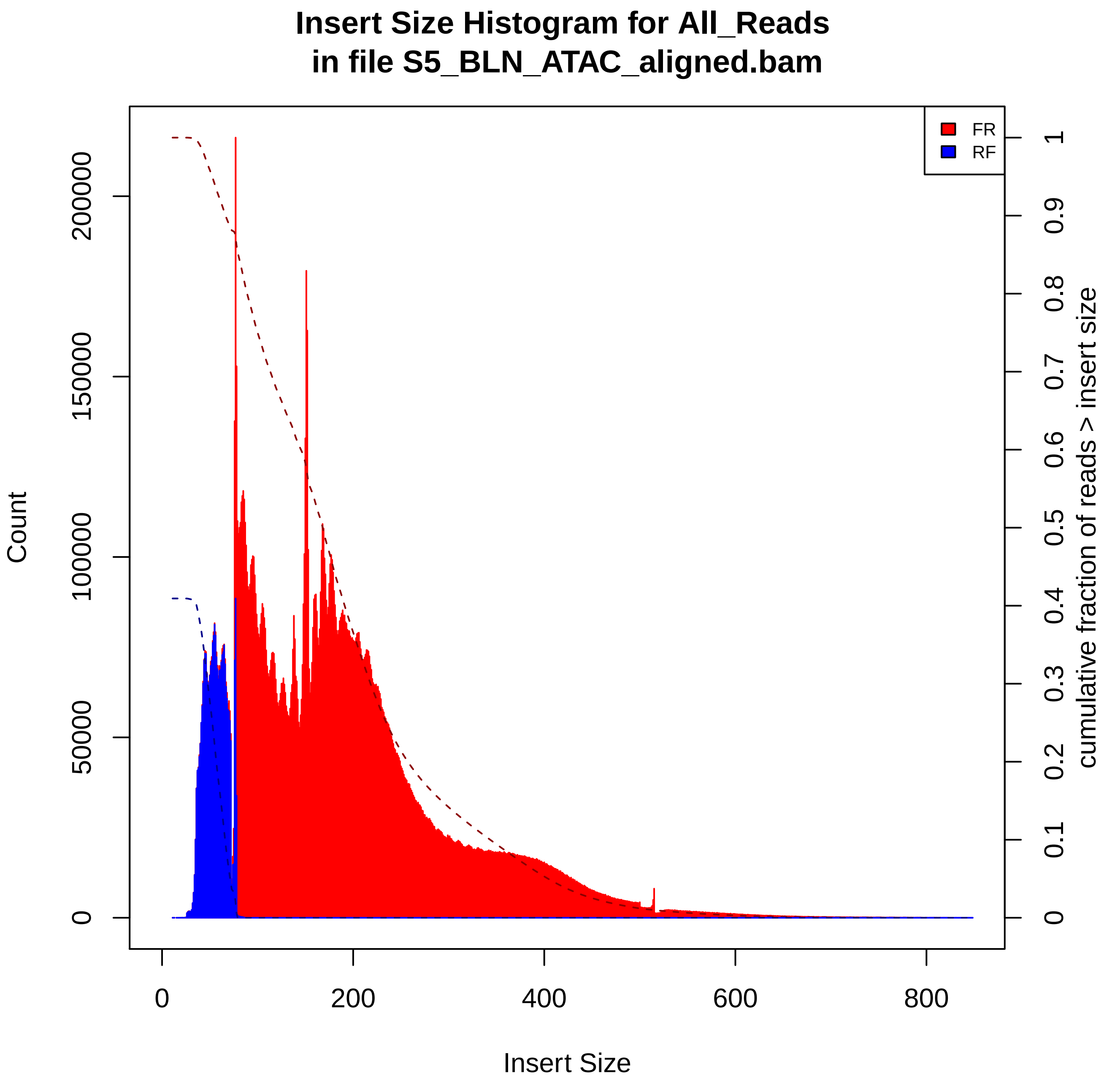
Insert Size Histogram for All_Reads
in file S5_BLN_ATAC_aligned.bam
FR
RF
Count
0
50000
100000
150000
200000
Insert Size
0
200
400
600
800
cumulative fraction of reads > insert size
0
0.1
0.2
0.3
0.4
0.5
0.6
0.7
0.8
0.9
1

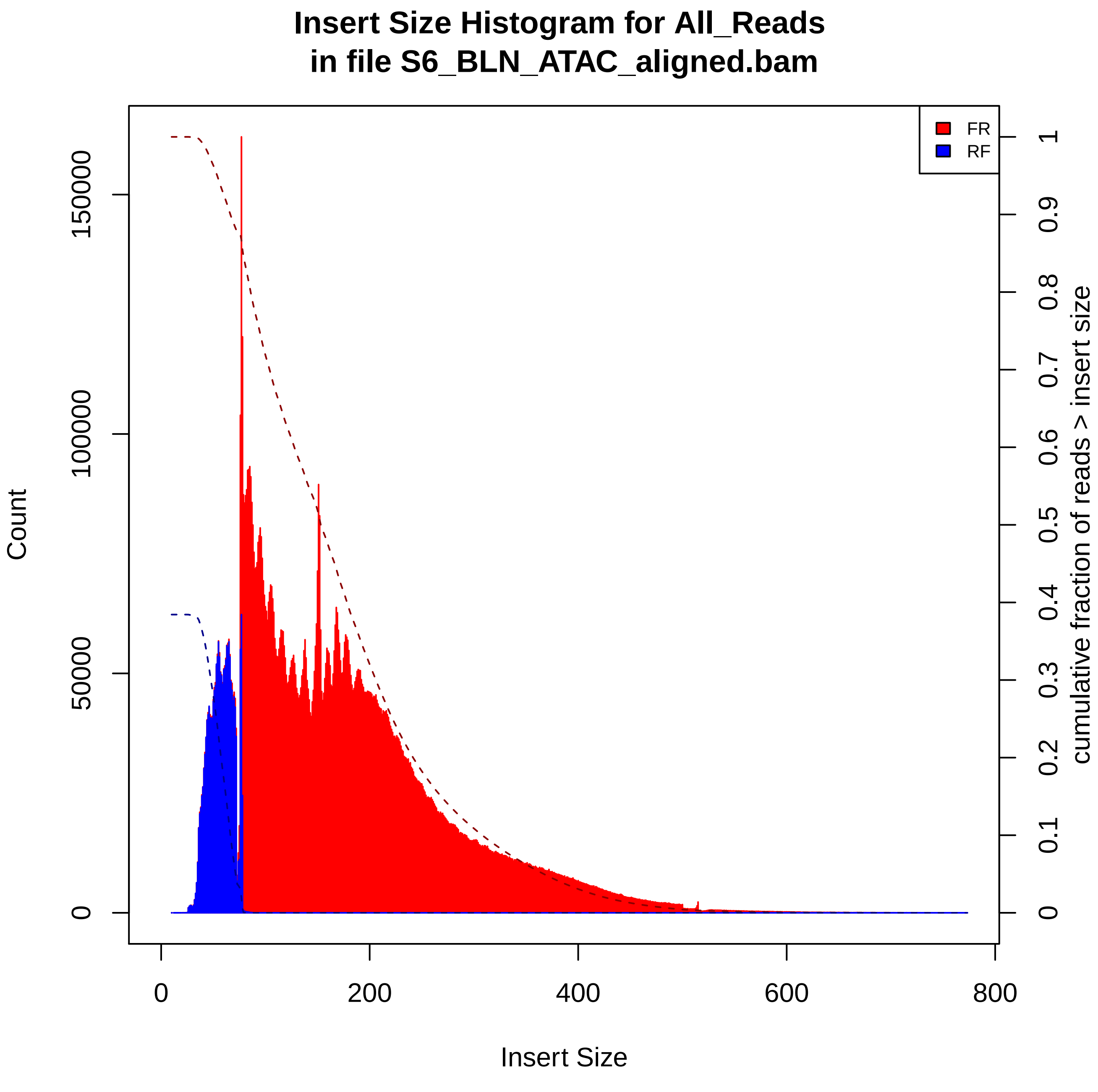

Insert Size Histogram for All_Reads
in file S6_BLN_ATAC_aligned.bam
Count
cumulative fraction of reads > insert size
Insert Size
FR
RF
0
50000
100000
150000
0
200
400
600
800
0
0.1
0.2
0.3
0.4
0.5
0.6
0.7
0.8
0.9
1

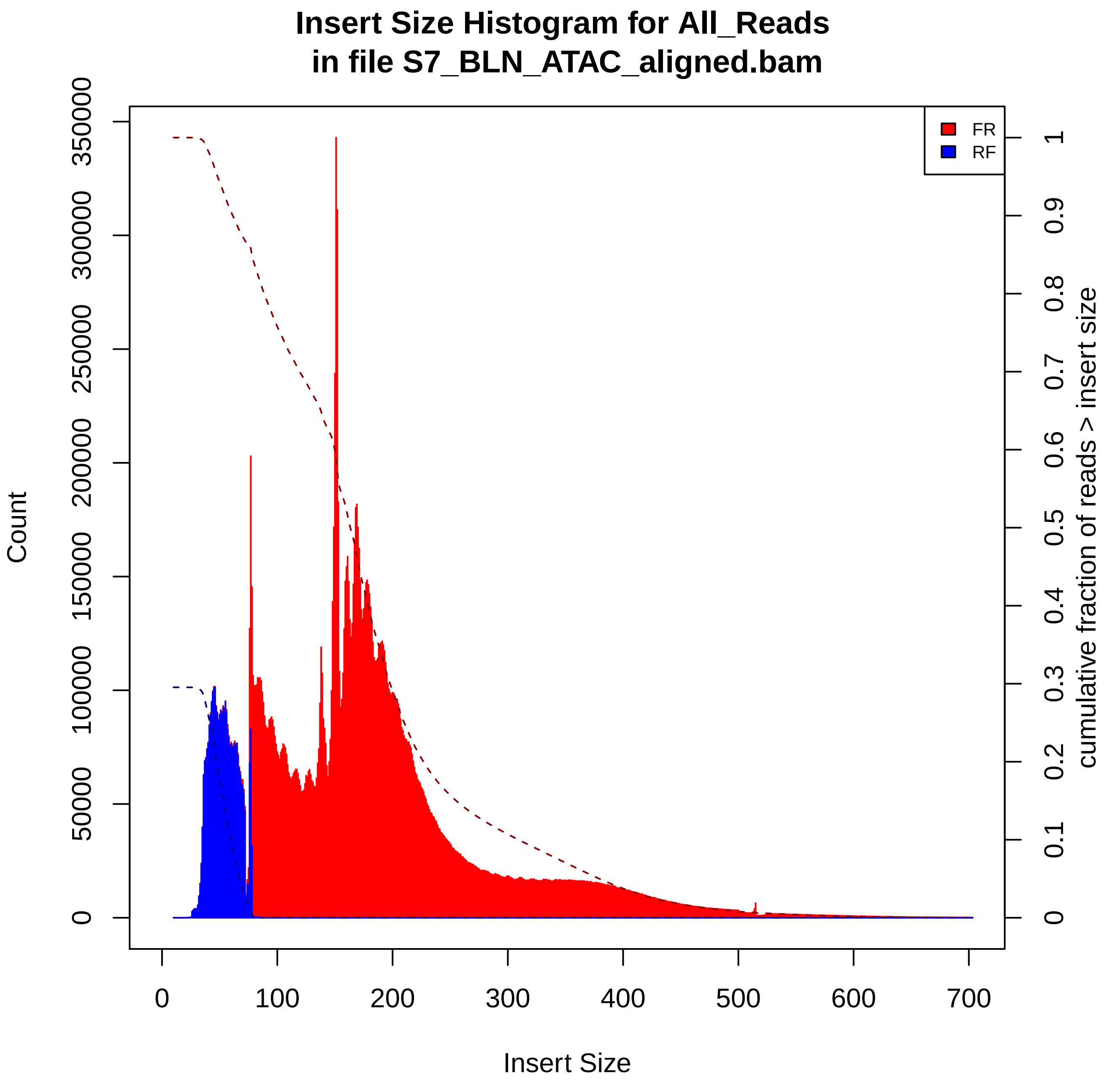
Insert Size Histogram for All_Reads
in file S7_BLN_ATAC_aligned.bam
Count
0
50000
100000
150000
200000
250000
300000
350000
Insert Size
0
100
200
300
400
500
600
700
cumulative fraction of reads > insert size
0
0.1
0.2
0.3
0.4
0.5
0.6
0.7
0.8
0.9
1
FR
RF

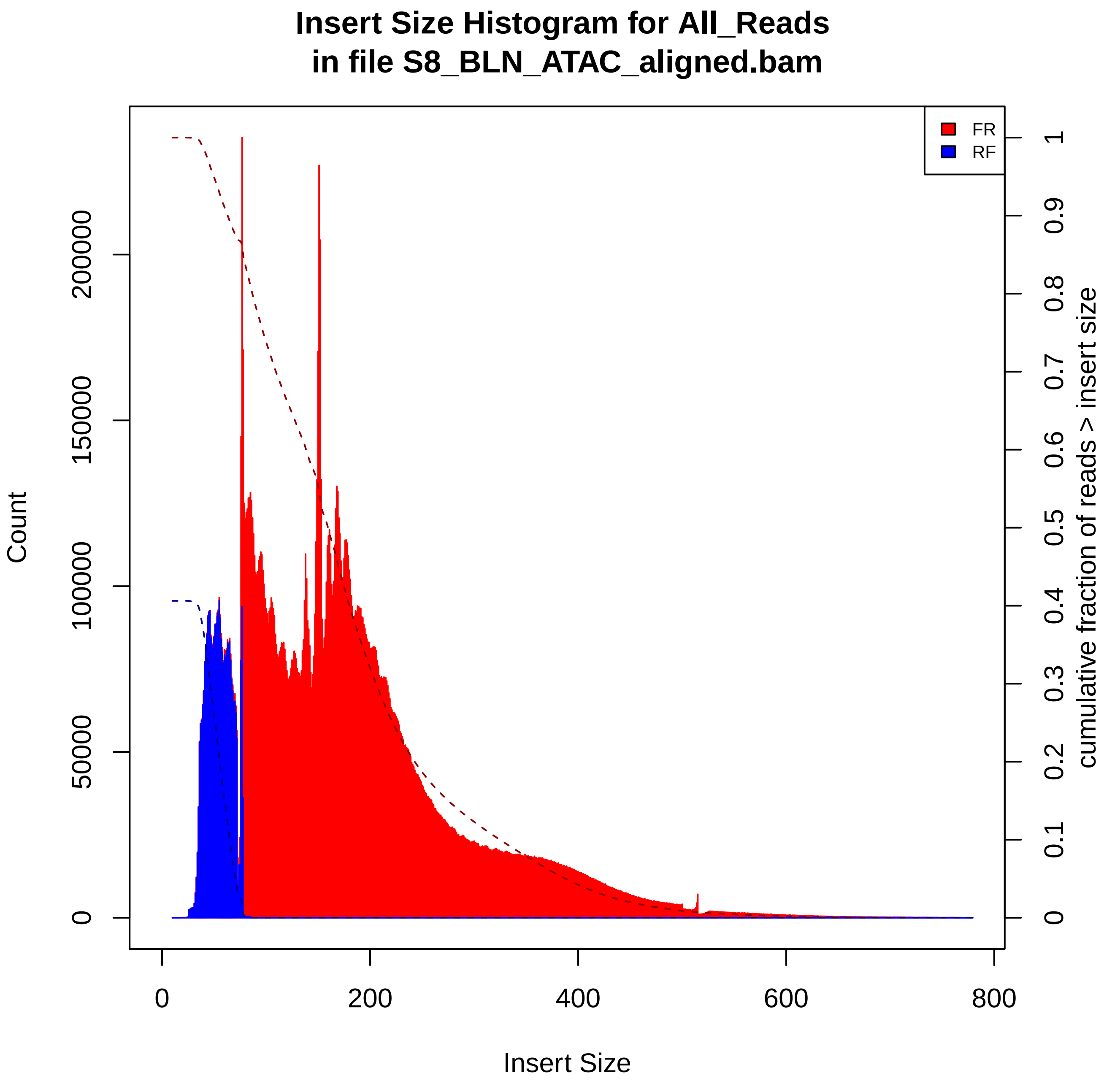

Insert Size Histogram for All_Reads
in file S8_BLN_ATAC_aligned.bam
FR
RF
Count
0
50000
100000
150000
200000
0
200
400
600
800
Insert Size
0
0.1
0.2
0.3
0.4
0.5
0.6
0.7
0.8
0.9
1
cumulative fraction of reads > insert size

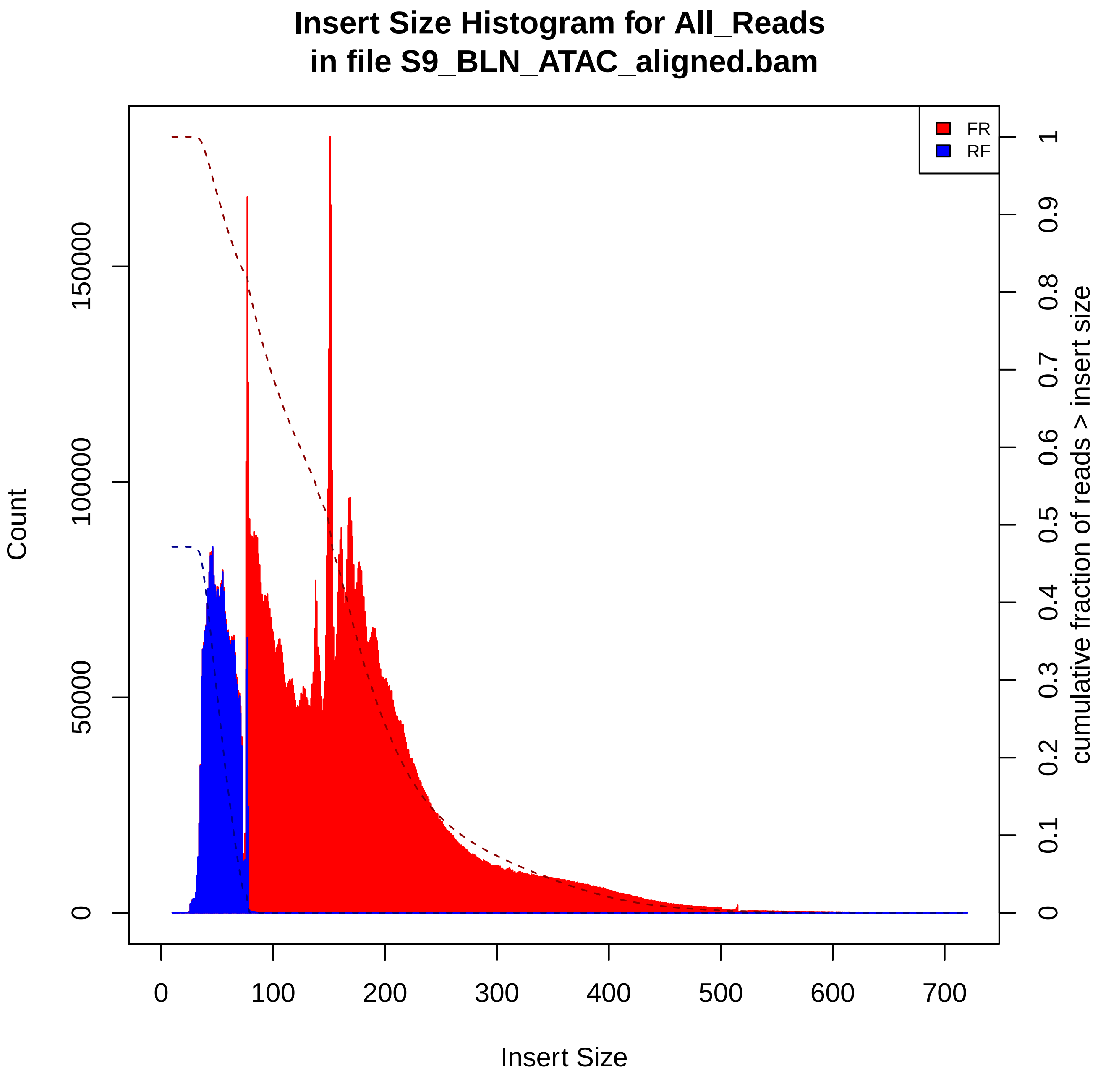

Insert Size Histogram for All_Reads
in file S9_BLN_ATAC_aligned.bam
FR
RF
Count
0
50000
100000
150000
0
100
200
300
400
500
600
700
Insert Size
0
0.1
0.2
0.3
0.4
0.5
0.6
0.7
0.8
0.9
1
cumulative fraction of reads > insert size

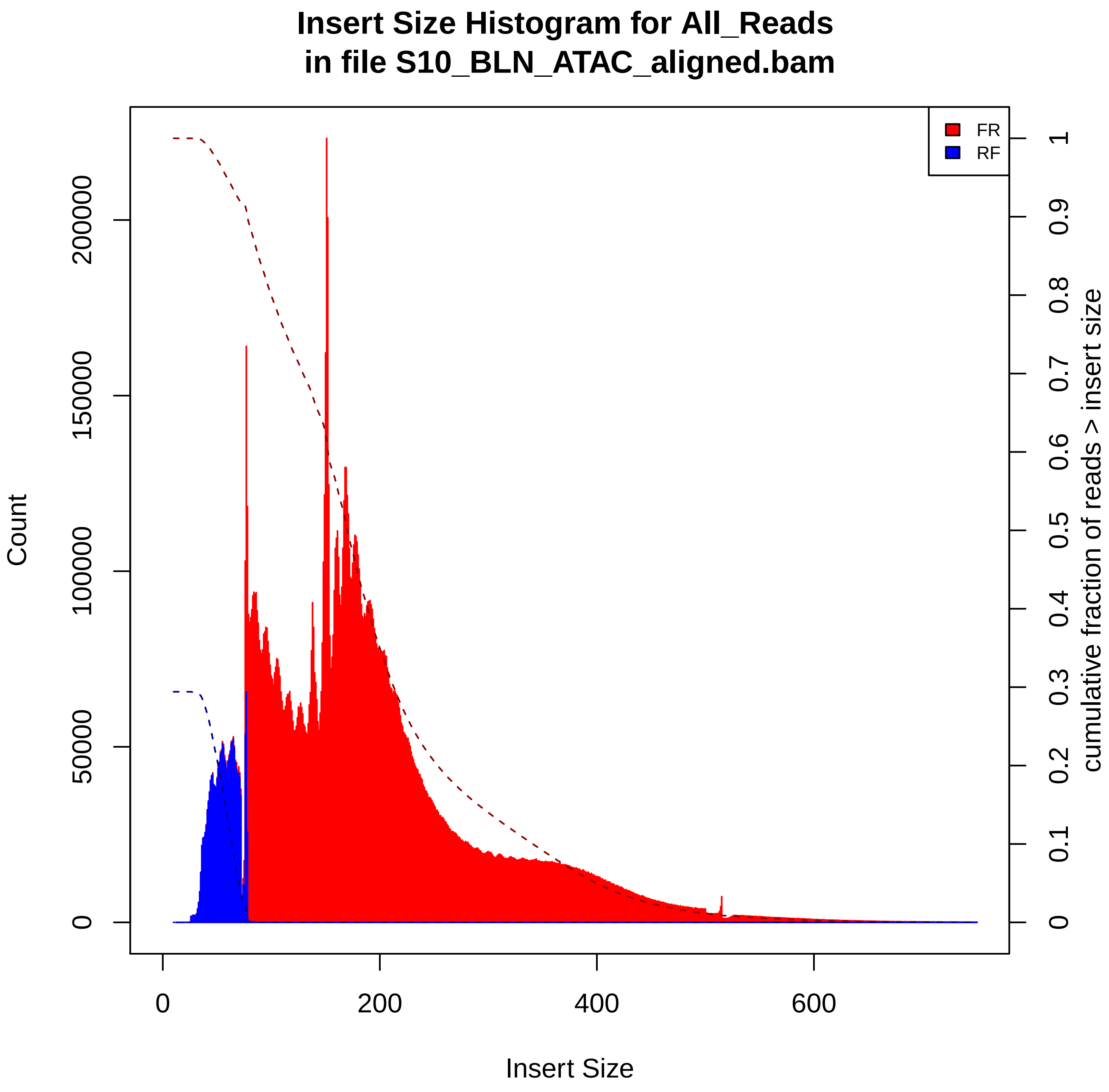

Insert Size Histogram for All_Reads
in file S10_BLN_ATAC_aligned.bam
FR
RF
Count
0
50000
100000
150000
200000
cumulative fraction of reads > insert size
0
0.1
0.2
0.3
0.4
0.5
0.6
0.7
0.8
0.9
1
Insert Size
0
200
400
600

Insert Size Histogram for All_Reads in file S11_BLN_ATAC_aligned.bam

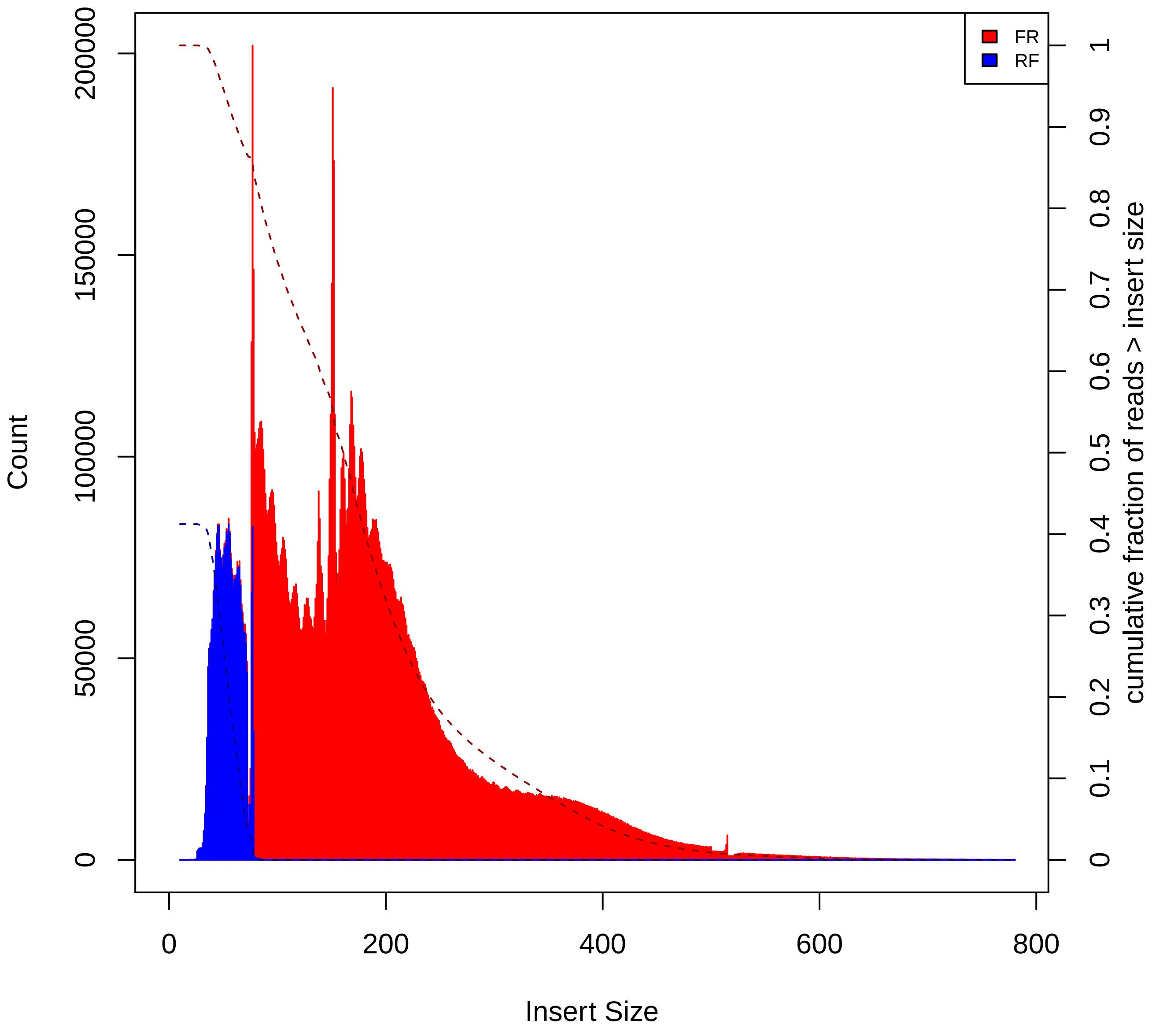

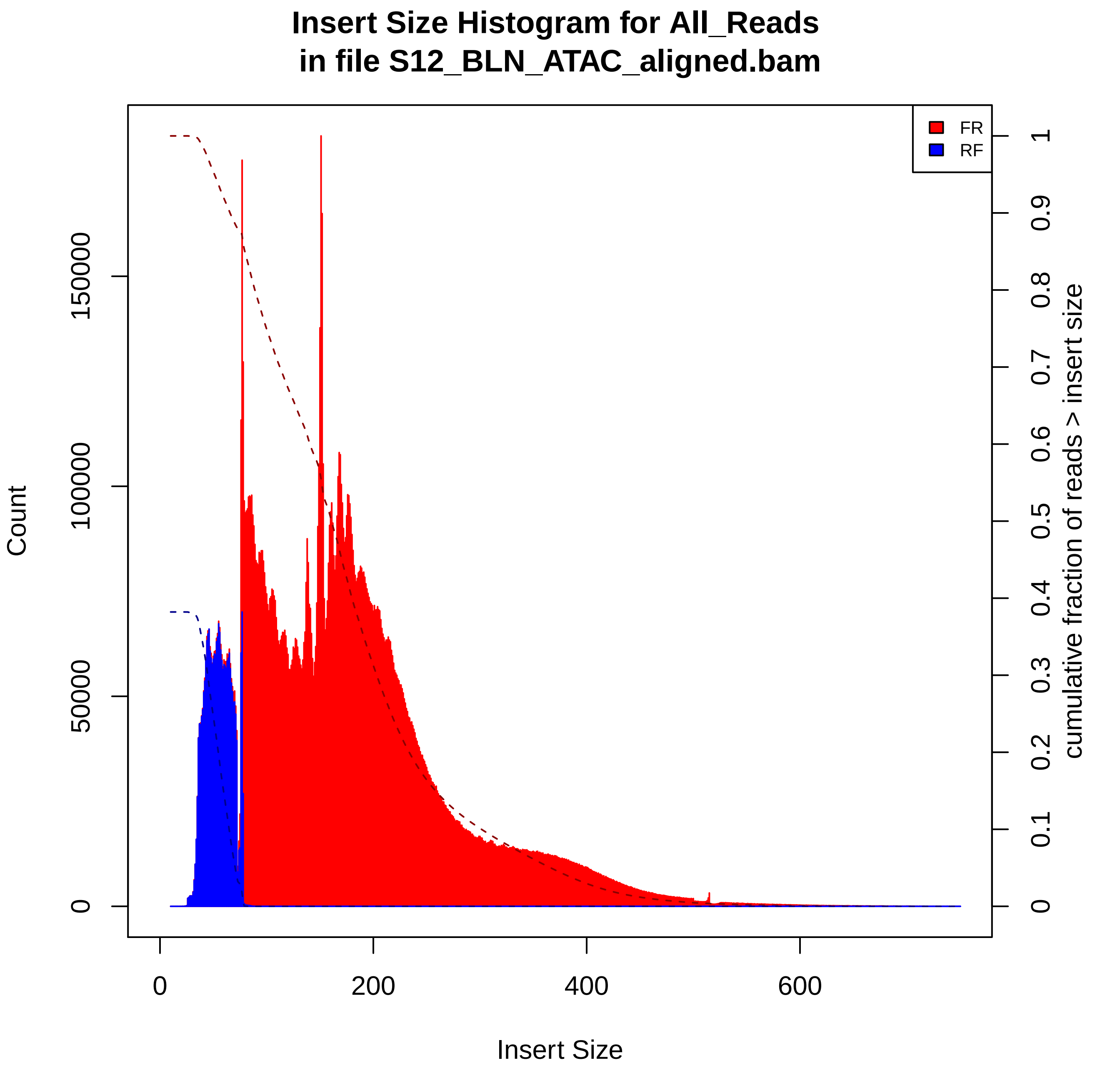
Insert Size Histogram for All_Reads
in file S12_BLN_ATAC_aligned.bam
FR
RF
Count
0
50000
100000
150000
0
200
400
600
Insert Size
cumulative fraction of reads > insert size
0
0.1
0.2
0.3
0.4
0.5
0.6
0.7
0.8
0.9
1

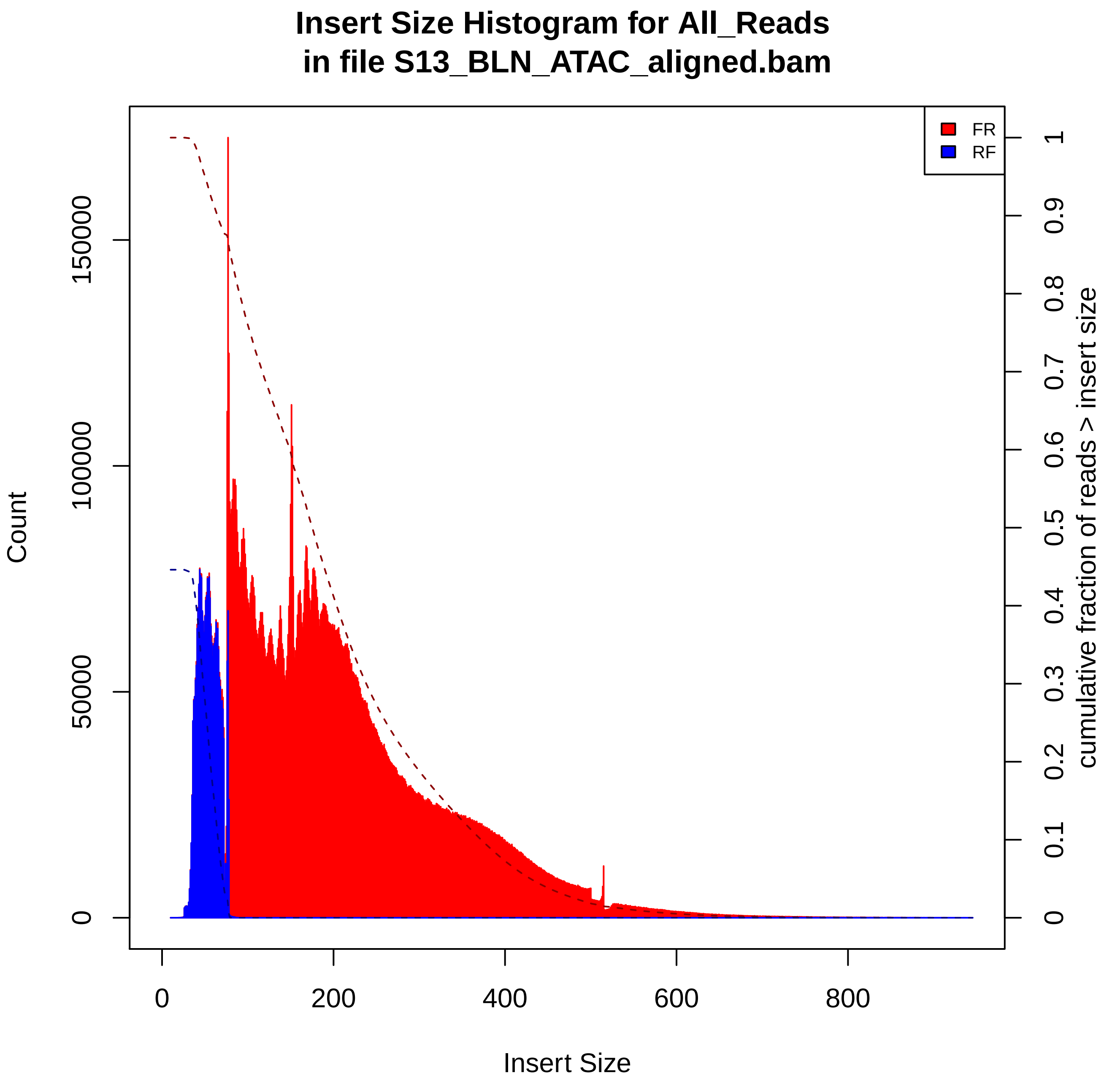

Insert Size Histogram for All_Reads
in file S13_BLN_ATAC_aligned.bam
FR
RF
Count
0
50000
100000
150000
0
200
400
600
800
Insert Size
cumulative fraction of reads > insert size
0
0.1
0.2
0.3
0.4
0.5
0.6
0.7
0.8
0.9
1

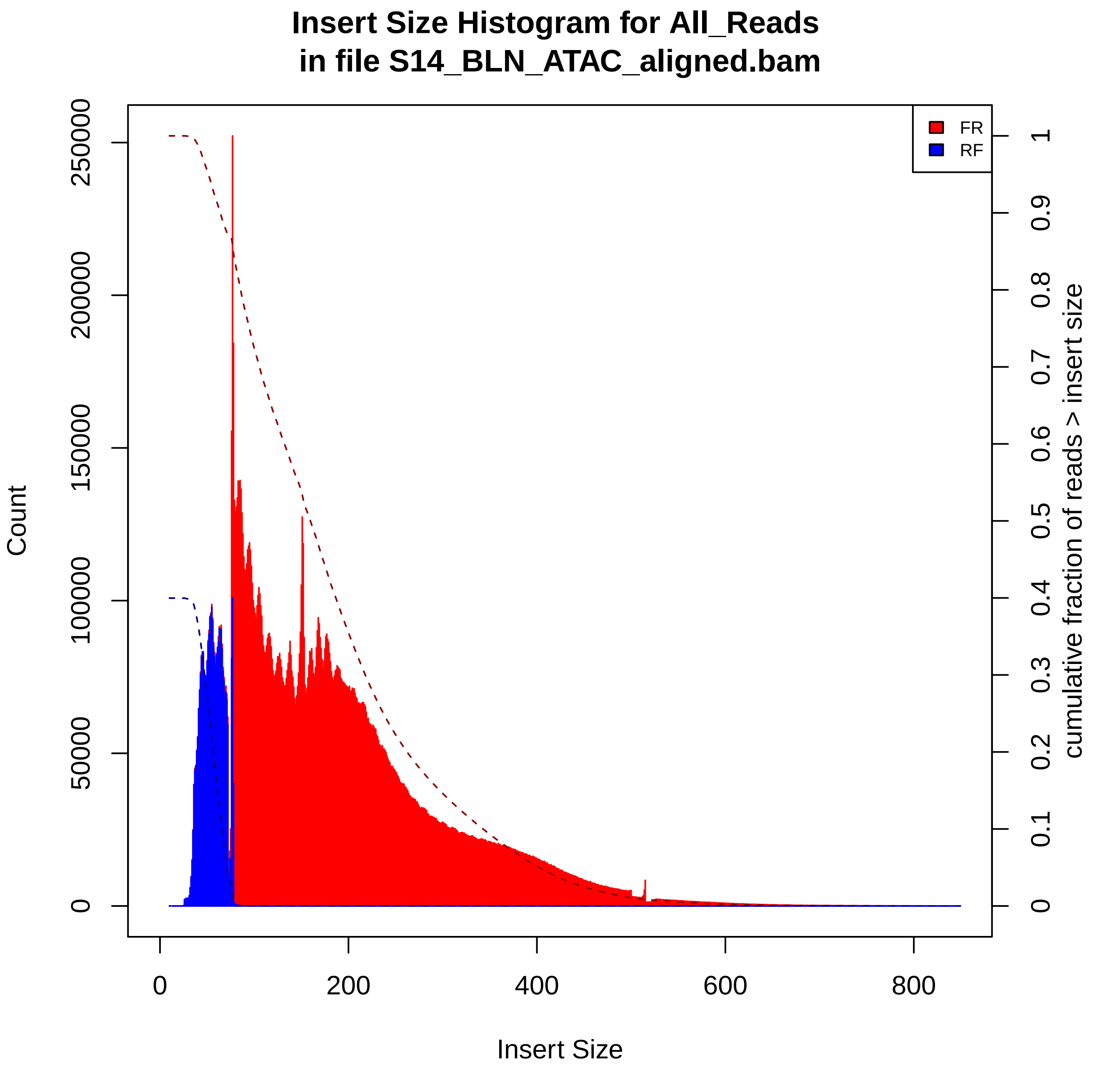

Insert Size Histogram for All_Reads
in file S14_BLN_ATAC_aligned.bam
FR
RF
Count
0
50000
100000
150000
200000
250000
cumulative fraction of reads > insert size
0
0.1
0.2
0.3
0.4
0.5
0.6
0.7
0.8
0.9
1
0
200
400
600
800
Insert Size

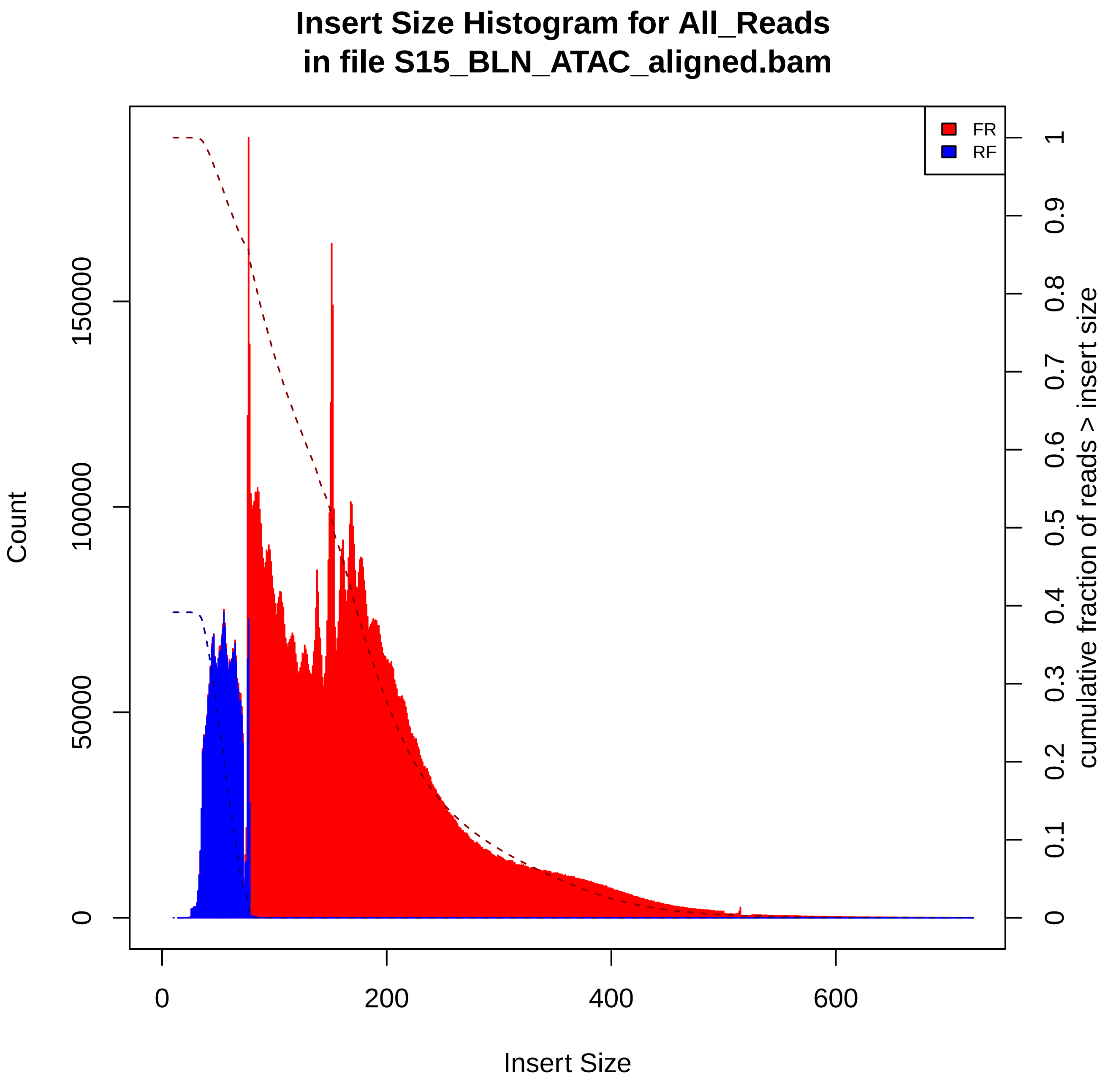

Insert Size Histogram for All_Reads
in file S15_BLN_ATAC_aligned.bam
Count
Insert Size
cumulative fraction of reads > insert size
FR
RF
0
50000
100000
150000
0
200
400
600
0
0.1
0.2
0.3
0.4
0.5
0.6
0.7
0.8
0.9
1

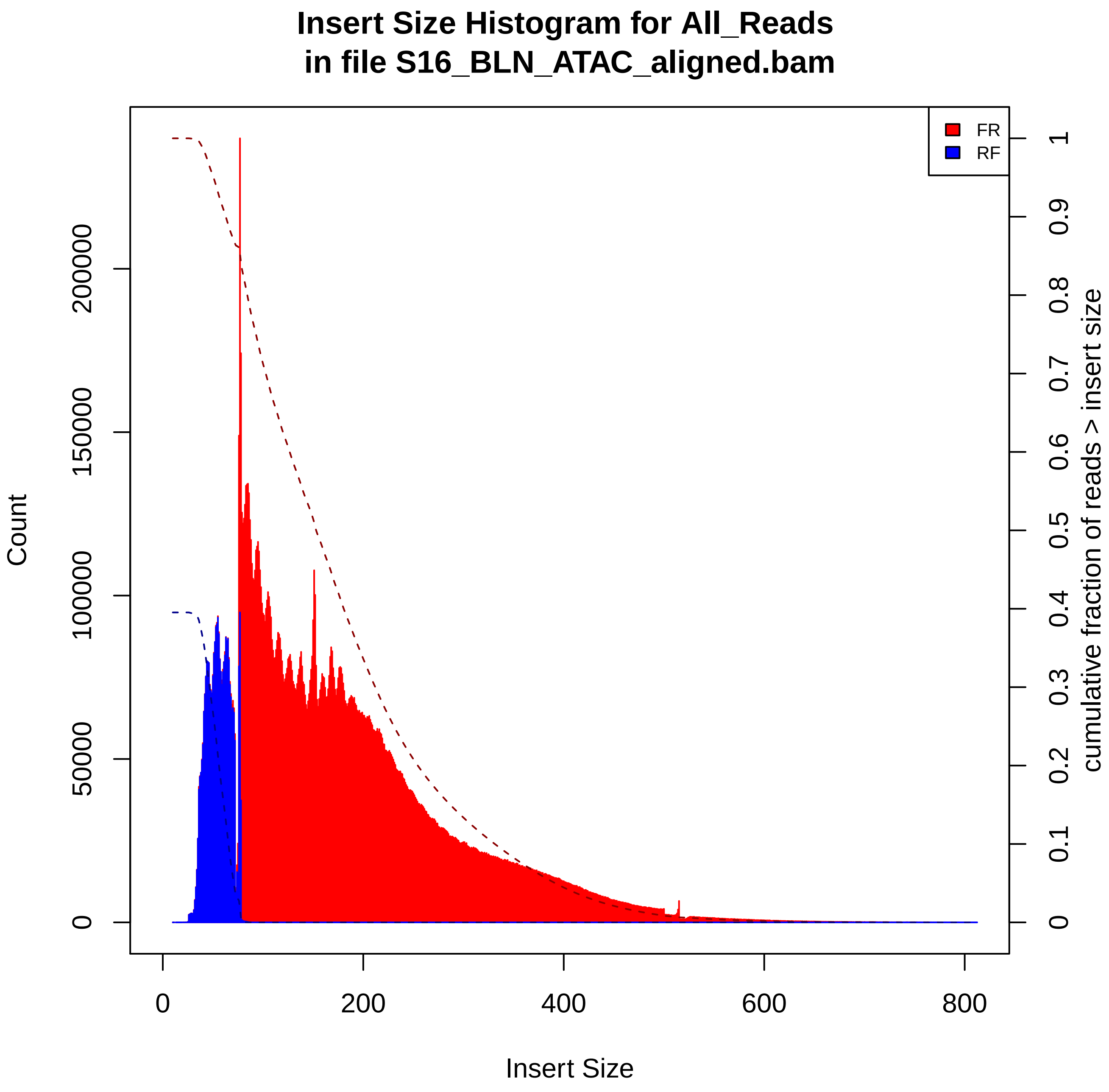

Insert Size Histogram for All_Reads
in file S16_BLN_ATAC_aligned.bam
FR
RF
Count
0
50000
100000
150000
200000
0
200
400
600
800
Insert Size
cumulative fraction of reads > insert size
0
0.1
0.2
0.3
0.4
0.5
0.6
0.7
0.8
0.9
1

Insert Size Histogram for All_Reads in file S17_BLN_ATAC_aligned.bam

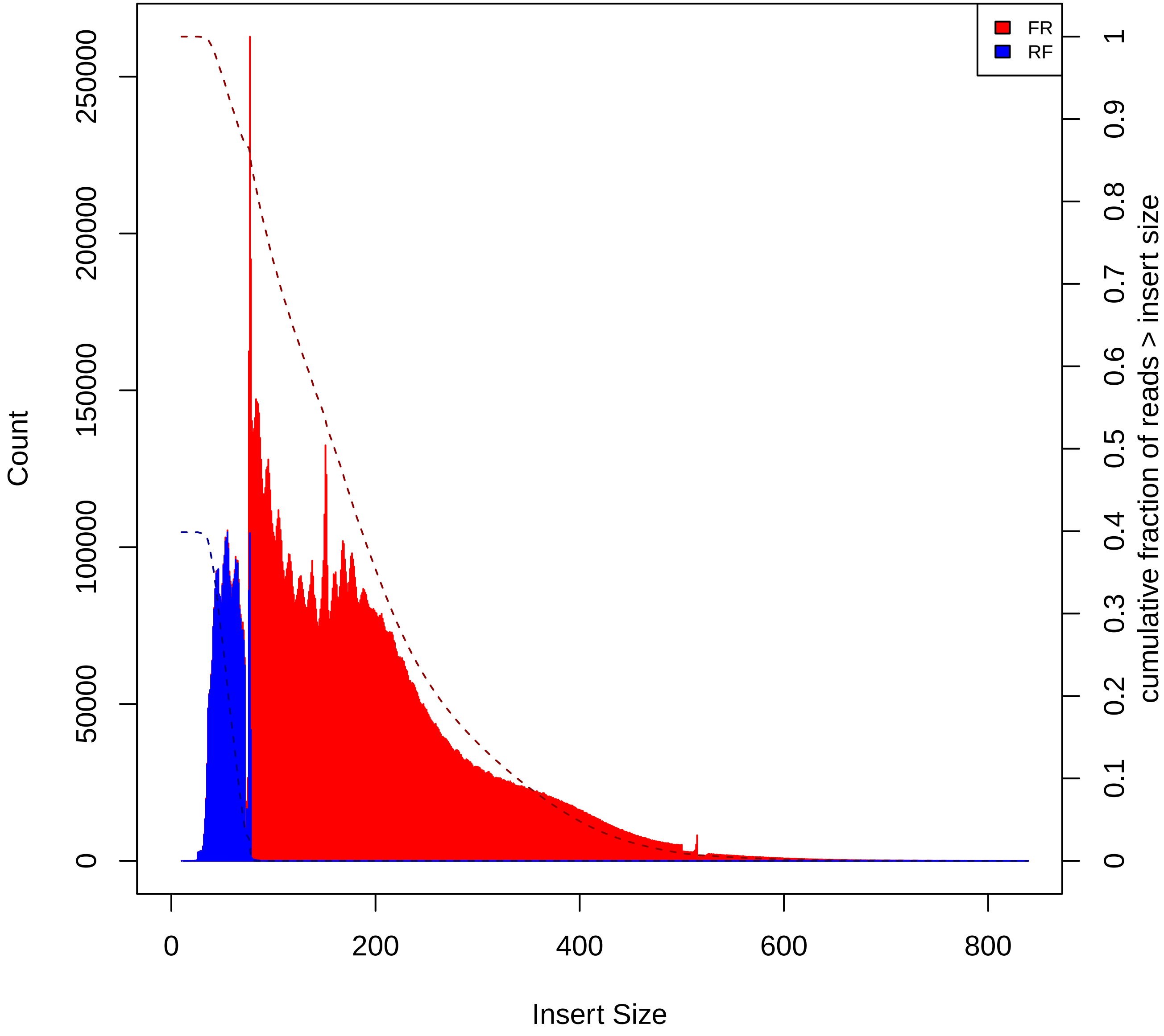

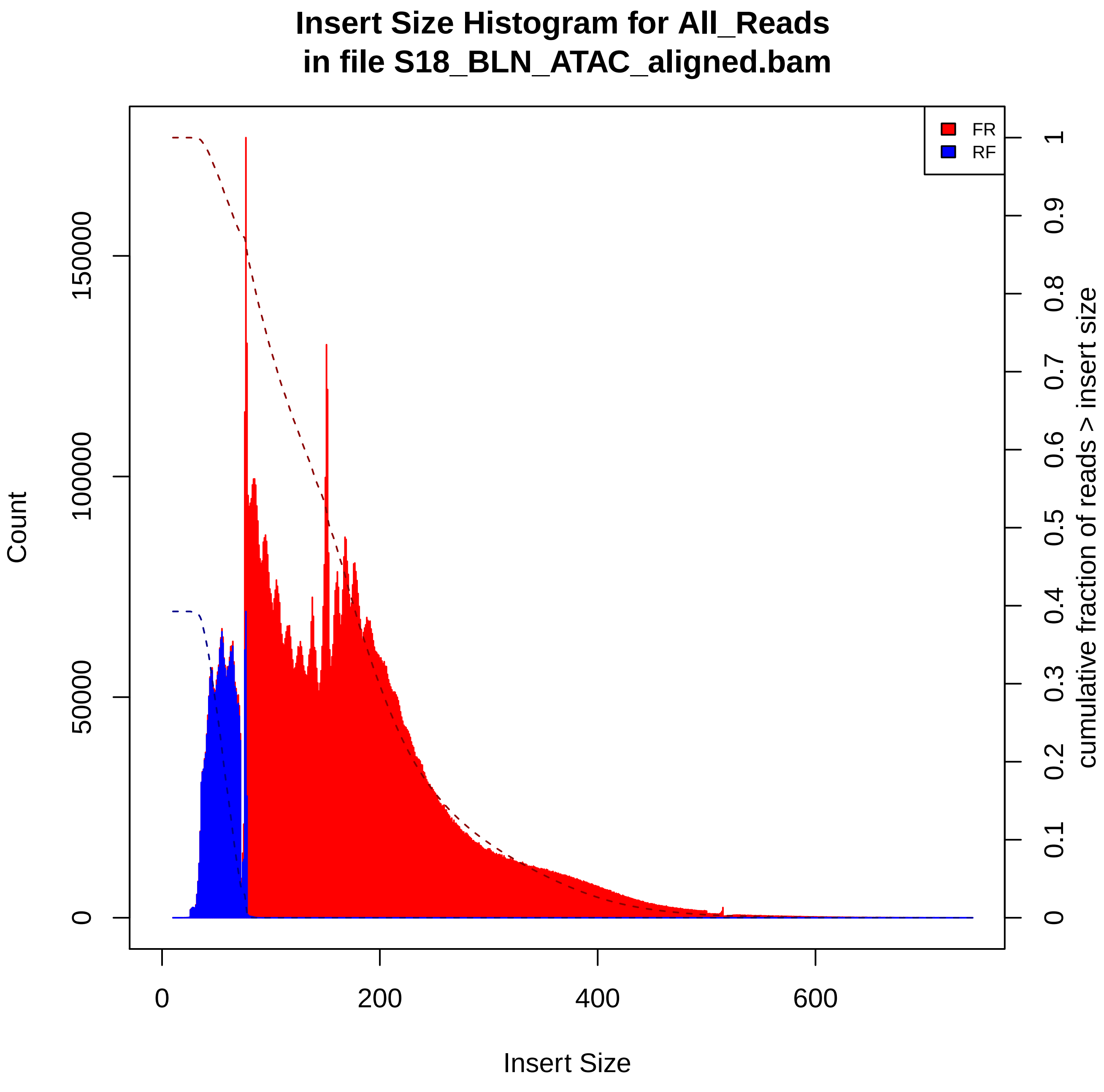

Insert Size Histogram for All_Reads
in file S18_BLN_ATAC_aligned.bam
FR
RF
Count
0
50000
100000
150000
0
200
400
600
Insert Size
0
0.1
0.2
0.3
0.4
0.5
0.6
0.7
0.8
0.9
1
cumulative fraction of reads > insert size
